# Efficient Screening of Coformers for Active Pharmaceutical Ingredient Cocrystallization

**DOI:** 10.1021/acs.cgd.2c00433

**Published:** 2022-06-15

**Authors:** Isaac J. Sugden, Doris E. Braun, David H. Bowskill, Claire S. Adjiman, Constantinos C. Pantelides

**Affiliations:** † Molecular Systems Engineering Group, Department of Chemical Engineering, Sargent Centre for Process Systems Engineering, Institute for Molecular Science and Engineering, Imperial College London, London SW7 2AZ, United Kingdom; ‡ University of Innsbruck, Institute of Pharmacy, Pharmaceutical Technology, Josef-Moeller-Haus, Innrain 52c, A-6020 Innsbruck, Austria

## Abstract

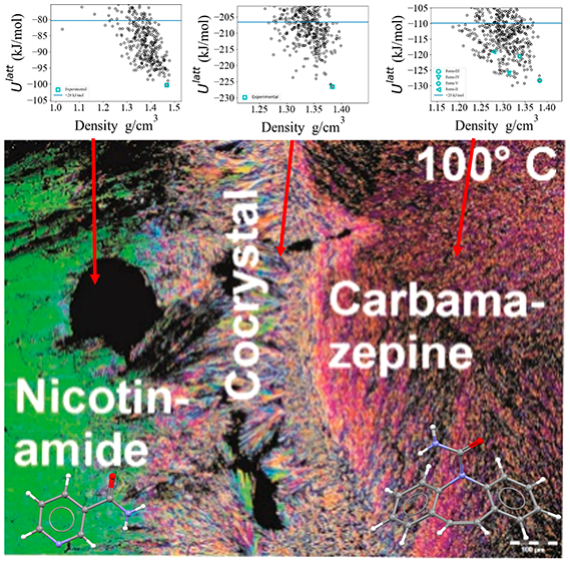

Controlling the physical properties of solid forms for active pharmaceutical ingredients (APIs) through cocrystallization is an important part of drug product development. However, it is difficult to know *a priori* which coformers will form cocrystals with a given API, and the current state-of-the-art for cocrystal discovery involves an expensive, time-consuming, and, at the early stages of pharmaceutical development, API material-limited experimental screen. We propose a systematic, high-throughput computational approach primarily aimed at identifying API/coformer pairs that are unlikely to lead to experimentally observable cocrystals and can therefore be eliminated with only a brief experimental check, from any experimental investigation. On the basis of a well-established crystal structure prediction (CSP) methodology, the proposed approach derives its efficiency by not requiring any expensive quantum mechanical calculations beyond those already performed for the CSP investigation of the neat API itself. The approach and assumptions are tested through a computational investigation on 30 potential 1:1 multicomponent systems (cocrystals and solvate) involving 3 active pharmaceutical ingredients and 9 coformers and one solvent. This is complemented with a detailed experimental investigation of all 30 pairs, which led to the discovery of five new cocrystals (three API–coformer combinations, a polymorphic cocrystal example, and one with different stoichiometries) and a *cis*-aconitic acid polymorph. The computational approach indicates that, for some APIs, a significant proportion of all potential API/coformer pairs could be investigated with only a brief experimental check, thereby saving considerable experimental effort.

## Abstract 2

Experimental and computational coformer screens are performed in parallel, for the combination of 3 active pharmaceutical ingredients and 10 coformers. The experimental screen generates five new cocrystal forms and one new pure form, while the computational methodology, which allows for reuse of quantum mechanical calculations, is demonstrated to have a dramatic increase in efficiency for studying cocrystals, if the pure forms have already been investigated.

## 1 Introduction

Multicomponent solid forms are important in the pharmaceutical industry as they allow increased control of the physical properties of an active pharmaceutical ingredient (API).^1−3^ Of particular importance are cocrystals, defined as “crystalline materials composed of two or more different molecular and/or ionic compounds generally in an integer stoichiometric ratio which are neither solvates nor simple salts”.^4^ The introduction of a cocrystallizing agent may be beneficial in cases where the available neat forms of an API have low solubility, potentially leading to low bioavailability and drug efficacy, as well as in cases where the neat forms are physically or chemically unstable, leading to issues in production.^5,6^ In such cases, a cocrystal may exhibit greater stability, thereby allowing the manufacture of a better drug product without compromising the therapeutic benefit of the API.

In the notorious case of Ritonavir,^7^ an anti-HIV drug on the World Health Organization’s list of “essential medicines”, a late-appearing polymorph with low solubility caused numerous formulation problems. The issue was resolved after four years of efforts by reformulating the API as a cocrystal with lopinavir. Cocrystals have increasingly been used in pharmaceutical products, including Depakote (valproic acid and sodium valproate), Cafcit (caffeine and citric acid), Lexapro (Escitalopram and oxalic acid), Odomzo (sonidegib and diphosphate), Suglat (proline and ipragliflozin), Entresto (sodium salts of sacubitril and valsartan), and Steglatro (ertugliflozin and l-pyroglutamic acid).^3^

Despite the potential contribution of cocrystals toward the development of improved drug products, the identification of suitable coformers for a given API remains a complex process. Experimental screening methods have been effective in several investigations,^8^ but screening of multiple API/coformer pairs can be time-consuming, expensive, and, in the early stages of a pharmaceutical development workflow, limited by a lack of sufficient amounts of API material. Thus, computational methods for the rapid and reliable assessment of the likelihood that a given API/coformer pair may form a stable cocrystal could significantly accelerate formulation development and mitigate the risks associated with the neat API solid form being unstable or insoluble. Given an API and a set of potential coformers (such as those in the Generally Regarded As Safe (GRAS) list^9^ or the Everything Added to Food list^10^), a computational screen would aim to determine which of these coformers are likely to lead to thermodynamically stable cocrystals for a given API. Those could then be studied experimentally to verify the existence or not of the predicted cocrystals.

Computational coformer screens presented in the literature have employed a number of different approaches. Some are based on the interactions of API/coformer dimers in the gas phase through calculations either of hydrogen bond propensity between API and coformer^11−13^ or of molecular electrostatic potential interactions^14^ through molecular complementarity methods.^15^ Further, the electron density of molecular components, combined with statistical thermodynamics in the COSMO-RS package,^16^ has been used to predict the cocrystallization propensity of selected APIs with libraries of coformers.^17^ Approaches based on machine learning methods using molecular descriptors^18^ have also been proposed. However, none of these methods consider explicitly the crystal environment and its effects on the stability of a proposed cocrystal. This deficiency can be addressed to some extent by crystal structure prediction (CSP) methods, the reliability of which has increased significantly^19,20^ over the past 30 years.

CSP methods have already been used successfully to study cocrystals in several investigations.^19,21−24^ Cocrystals are particularly challenging in this context because introducing more molecules into the crystal’s asymmetric unit increases the number of degrees of freedom that need to be explored in identifying all low-energy crystal structures. Thus, the *de novo* generation of a computational landscape for a given pair of compounds with state-of-the-art methods can often take hundreds of thousands or even millions of CPU hours.^25,26^

Another challenge for the application of CSP methods to cocrystals relates to the derivation of sufficiently accurate lattice energy models (force fields) for a relatively large number of API + coformer pairs. In some CSP methods, such as GRACE^27^ or XtalPi,^28^ the force field is tailored to the system under consideration by carrying out periodic dispersion-corrected density functional theory (DFT-d) calculations^29^ on a set of crystal structures. Thus, for each API + coformer pair, an initial set of cocrystal structures must be generated, followed by an expensive DFT-d calculation for each such structure. By contrast, in the CrystalPredictor^30^/CrystalOptimizer^31^ framework, isolated-molecule *in vacuo* quantum mechanical (QM) calculations are carried out separately for each component of the crystal. The results of such QM calculations at different molecular conformations are used to construct local approximate models (LAMs)^31^ that can compute the intramolecular energy (Δ*U*^intra^) and electronic density (in terms of atomic charges and multipoles) as explicit, computationally cheap functions of molecular conformation. For moderate changes in molecular conformation around the reference point at which the corresponding QM calculations are performed, it has been shown that it is possible to derive a set of LAMs that collectively can provide QM-like accuracy in describing intramolecular energy, electrostatic potential, and geometry.^32^

LAMs are used at both the global search stage of a CSP investigation^33−35^ and at the refinement stage.^31^ In the former case, they are constructed *a priori* at selected sets of points organized in uniform or nonuniform grids over the molecular conformational space. At the refinement stage, they are constructed “on the fly” as new areas of conformational space are explored by the lattice energy minimization algorithm. At both stages, LAMs are stored in a database from where they can be retrieved in order to avoid repeating QM calculations at the same or similar molecular conformations.

Because of the computational cost of the QM calculations, constructing the LAM database is typically the most expensive element of a CSP investigation. However, in the context of applying CSP to cocrystal screening, the above approach can lead to dramatic cost reductions by taking advantage of the fact that a given compound’s LAM database is independent of crystal structure or of any other compound in the lattice. Thus, for a given API, the *same* LAM database can be used in assessing the likelihood of cocrystal formation against multiple coformer candidates. Moreover, since the set of acceptable coformers for applications such as pharmaceuticals is fixed, the LAM database for each one of these coformers can be constructed once and for all, for subsequent use with any API of interest.

Building on the above ideas, in this paper, we propose a systematic and efficient approach for performing a computational coformer screen for a given API and a set of candidate coformers, based on multiple CSP investigations carried out in parallel. We test the reliability of this approach by also carrying out experimental coformer screens using a variety of approaches (contact preparation method, slurry experiments, liquid-assisted grinding, solvent evaporation, and cosublimation) chosen to achieve a thorough search and one that could be carried out in an industrial context.

The approaches used for studying cocrystals computationally and experimentally are outlined in sections 2.1 and 2.2, respectively. The algorithm for determining if a cocrystal is likely to form between an API and a given coformer is outlined in section 3. In section 4, we demonstrate the technique through a case study involving three APIs and a set of nine coformers and a solvent from the GRAS list. The computational results are compared to those of the experimental screening.

## 2 Methods

For the purpose of this article, we will consider standardized computational and experimental methods that are accurate yet affordable, allowing them to be adopted within the context of current pharmaceutical practices. These are outlined below.

### 2.1 Computational Method

CSP calculations for all systems are carried out using the code CrystalPredictor version 2.4.3.^35^ The flexible conformational degrees of freedom are determined based on the changes in intramolecular energy values arising from ±15° perturbations applied to those torsional angles that were identified as potentially flexible by the values of second derivatives at the gas-phase conformational minimum. Isolated-molecule QM calculations are performed in Gaussian 09 at the PBEPBE level of theory using the 6-31G(d,p) basis set. Starting with an initial uniform LAM grid, the adaptive LAM algorithm^32^ is run until convergence is achieved, with the convergence criterion Δ* equal to 5 kJ/mol. The set of parameters referred to as the “FIT potential” is used to describe the exchange-repulsion and dispersion interactions.^36−39^ In the global search space, 500 000 structure minimizations are performed, sampling the 59 most common space groups.

After the CrystalPredictor calculations are completed, a final clustering of generated structures is carried out with the COMPACK algorithm. All unique structures within 20 kJ/mol (to a maximum of 500 structures) of the global lattice energy minimum from this process are refined using CrystalOptimizer 2.4.5. The latter makes use of the results of QM calculations performed at the PBE1PBE/6-31G(d,p)) level of theory; additional flexibility is introduced (all angles involved in previously determined torsions), and electrostatic interactions are described by atomic multipoles. Repulsive/dispersive interactions are described by the DB2021 set of parameters, which were fitted to reproduce periodic DFT geometries and energies across an extensive set of crystal structures of organic molecules, at the same level of theory.^40^

### 2.2 Experimental Method

#### 2.2.1 Materials

Work was done on commercially available samples (purity > 98%) without further purification: carbamazepine (CBZ, G.L. Pharma), acetylsalicylic acid (aspirin, Gatt-Koller), acetaminophen (paracetamol, Bayer), pyridoxine (Sigma), methyl paraben (Merck), propyl 4-hydroxybenzoate (Merck), 3-*t*-butyl-4-hydroxyanisole (Aldrich), nicotinic acid (Bayer), nicotinamide (Merck), oxalic acid (Merck), succinic acid (Fluka), and *cis*-aconitic acid (Aldrich). Pyridine, chloroform, and *n*-heptane were of p.a. grade (>99.8% pure) and purchased from Merck or VWR.

#### 2.2.2 Contact Preparation Method/Hot-Stage Thermomicroscopy (HSM)

Where the thermal stability and relative differences in melting points allowed, the contact preparation method was used to investigate cocrystal formation. An Olympus BH2 polarization microscope (Olympus Optical GmbH, Vienna, Austria) equipped with a Kofler hot-stage (Reichert Thermovar, Vienna, Austria) was used. Cocrystals were prepared on the hot-stage by first melting the higher-melting compound on a microscopic slide covered by a glass slide and subsequently cooling it down to create a thin crystal film. The lower-melting compound was then melted on the same microscopic slide. Through capillary action, the liquid was drawn below the cover slide until it reached the higher-melting coformer and then rapidly cooled. This gave rise to the possibility of a crystalline film of the cocrystal forming at the zone of mixing.

#### 2.2.3 Slurry Experiments

Mixtures of a 1:1 molar ratio were prepared and transferred to small vials. An amount of 300–600 μL of solvent was added to 200 mg of this mixture, and the slurry was stirred in the temperature range from 10 to 30 °C for up to 4 weeks. Samples were drawn periodically, for the first week on a daily basis, and then every week, and analyzed with PXRD.

#### 2.2.4 Liquid-Assisted Grinding (LAG)

Mixtures of a 1:1 molar ratio were prepared and thoroughly ground using an agate mortar and pestle. One to two drops of solvent were added to an amount of 100 mg, and the paste was subsequently milled in five stainless steel vessels with two balls of the same material and 0.5 cm in diameter using a Retsch ball mill MM 301 (Haan, Germany) at 30 Hz for 15 min. The resulting powders were analyzed with IR spectroscopy and PXRD.

#### 2.2.5 Solvent Evaporation

1:1 molar mixtures of the APIs and coformers were dissolved at room temperature and transferred into vials with pierced lids, and then the solvent was allowed to slowly evaporate.

#### 2.2.6 Cosublimation Experiments

Equimolar amounts were prepared, thoroughly ground using an agate mortar and pestle, and transferred into 0.3 mL vials. The sublimation experiments were carried out under elevated temperatures at a pressure of 200 mbar with the aid of a CrystalBreeder (Technobis, The Netherlands).

#### 2.2.7 Powder X-ray Diffraction

PXRD patterns were obtained using an X’Pert PRO diffractometer (PANalytical, Almelo, The Netherlands) equipped with a θ/θ coupled goniometer in transmission geometry, programmable XYZ stage with a well plate holder, Cu–K_α1,2_ radiation source with a focusing mirror, a 0.5° divergence slit, a 0.02° Soller slit collimator on the incident beam side, a 2 mm antiscattering slit, a 0.02° Soller slit collimator on the diffracted beam side, and a solid-state PIXcel detector. The patterns were recorded at a tube voltage of 40 kV and tube current of 40 mA, applying a step size of 2θ = 0.013° with 40 or 80 s per step in the 2θ range between 2° and 40°.

#### 2.2.8 Pawley Fitting and Rietveld Refinements

Selected diffraction patterns were indexed with DICVOL04 using the first 12–20 peaks, and the space group was determined based on a statistical assessment of systematic absences,^41^ as implemented in the DASH structure solution package.^42^ Pawley fits^43^ and Rietveld refinements^44^ were performed with Topas Academic V5.^45^ Simulated annealing was used to optimize PBE-TS models against the diffraction data sets in direct space. The internal coordinate (Z-matrix) descriptions of the molecules were derived from computed structures (see section 3.1 of the Supporting Information). The structures were solved using 100 simulated annealing runs of 2.5 × 10^8^ moves per run as implemented in DASH, allowing external and internal degrees of freedom. The best solutions returned were used as a starting point for PBE-TS fixed unit cell structure optimizations using CASTEP.^46^ The optimized structures were then used as the starting points for rigid body Rietveld refinements in Topas. For more details, see section 3.5 of the Supporting Information.

## 3 Proposed Approach to Computational Coformer Screening

Given an API and a set of candidate coformers *c* = 1, ..., *N*, we assess the likelihood of a cocrystal of stoichiometry API_*n*_*c*_*m*_ being observed experimentally by considering the free energy difference quantity ΔΔ*G*_*c*_ defined as
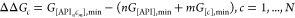
1where *G*_[API_*n*_*c*_*m*_],min_ is the free energy of the most stable cocrystal structure, while *G*_[API],min_ and *G*_[*c*],min_ are the free energies of the most stable neat-crystal structures of the API and coformer, respectively. In practice, and given the established expense of computing free energies of crystal lattices,^47,48^ we replace the free energy quantities with lattice energies at 0 K, leading to the quantity:
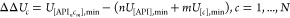
2Equation 1 assumes that the API and the coformer *c* are both solids at the temperature (e.g., room temperature, 300 K) at which the solid cocrystal API_*n*_*c*_*m*_ is considered. For cases where the coformer *c* is liquid at this temperature, the cocrystal is effectively a solvate, and the last term of eq 1 needs to be augmented by the coformer’s free energy of fusion Δ*G*_[*c*]fus_ at this temperature:
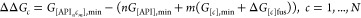
3By considering an appropriate thermodynamic cycle, Δ*G*_[*c*]fus_ can be related to the coformer’s enthalpy of fusion Δ*H*_*c*,fus_ at the melting point *T*_*c*,fus_ via the equation:^49^
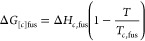
4Once again, replacing solid-state free energies with lattice energies at 0 K leads to the equation:
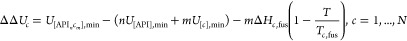
5In general, the less negative ΔΔ*U*_*c*_, the less likely it is for the API to form a cocrystal with coformer *c*.

Our proposed screening methodology is based on the following algorithm:

(1) If not already available, develop a LAM database for API by performing a CSP study on that molecule.
(2) Evaluate lattice energy *U*_[API],min_ for *either* the lower-energy structure determined by the CSP study at step 1 *or* the most stable structure that has been observed experimentally.
(3) For each coformer *c* = 1 to *N*:

(a) If not already available, develop a LAM database for coformer *c* by performing a CSP study on that molecule.
(b) Evaluate lattice energy *U*_[*c*],min_ for *either* the lower-energy structure determined by the CSP study at step 3a *or* the most stable structure that has been observed experimentally.
(c) For all cocrystal stoichiometries *m*/*n* of interest, perform a CSP study on cocrystal API_*n*_*c*_*m*_ to determine the most stable cocrystal structure and the corresponding lattice energy *U*_[API_*n*_*c*_*m*_],min_.
(d) Compute quantity ΔΔ*U*_*c*_ from eq 2 (or 5 if solvate).

(4) Create a ranked list of all candidate coformers sorted in order of increasing ΔΔ*U*_*c*_.

We note that similar methodologies have also been used in recent investigations into three-component ionic cocrystals,^50^ solvates (with the energy costs for converting a liquid solvent molecule to a solid accounted for with a 3/2 *RT* term),^51^ and hydrates.^52,53^ A similar approach has also been undertaken with DFT-d methods and proven successful.^25^ However, the key advantage of the proposed approach is its computational efficiency arising from the use of the CrystalPredictor^30^/CrystalOptimizer^31^ framework for the CSP studies at steps 1, 3a, and 3c of the algorithm in an integrated manner.

More specifically, the main computational cost of the CrystalPredictor/CrystalOptimizer framework is that of isolated-molecule quantum mechanical calculations (cf. section 1); as an example, two carbamazepine structures minimized using CrystalOptimizer without LAM databases cost on average 32 CPU hours, while it costs 8 CPU hours if the database is already populated. In comparison, using periodic DFT (VASP) and the TPSS functional and tight settings, it costs approximately 10,000 CPU hours to minimize one *Z* = 4 structure (the similarly sized Pyridoxine experimental form). The proposed algorithm derives its efficiency from minimizing the need for such calculations. In particular,

- The CSP study of step 1 will typically have been performed already in the context of studying the neat API crystal structures.
- The CSP study at step 3a will need to be performed once only for each individual coformer *c*. The LAM database generated by it can then be stored to be reused in future screenings involving this coformer in the context of *any* API.
- The CSP studies at step 3c make use of the LAM databases already obtained at steps 1 and 3a. No new quantum mechanical calculations are necessary.

For application areas such as pharmaceuticals, one can envisage a once-only effort to obtain a set of LAM databases covering all coformers of potential interest. From that point onward, it will be possible to perform the computational coformer screening for any new API with relatively little additional cost beyond that involved in the CSP study of the neat API itself.

We note that the quantities *U*_[API],min_ and *U*_[*c*],min_ may be computed for the most stable crystal structure determined either by the corresponding CSP studies or experimentally (cf. steps 2 and 3b respectively). The choice between these two options will depend on the user’s assessment of the relative reliability of computational versus experimental information. For molecules that have already been the subject of extensive experimental studies, the experimentally observed structures may be more reliable. On the other hand, for relatively new molecules (e.g., a new API that is still under development), it may be preferable to use the most stable structures identified by the CSP study.

The final result of the proposed algorithm is a list of all candidate coformers *c* ordered in increasing ΔΔ*U*_*c*_ (cf. step 4 of the algorithm). Assuming a negative correlation between ΔΔ*U*_*c*_ and the likelihood of being able to obtain the corresponding cocrystal, this list can inform a prioritized program of cocrystallization experiments to determine whether the predicted cocrystals can actually be identified in practice. A particularly useful objective in this context would be to establish a *maximum* value ΔΔU* beyond which cocrystals are very unlikely to be observed, thereby allowing some of the candidate coformers to be eliminated from the experimental program. One recent systematic review of known cocrystals suggested that ΔΔU* is on the order of 10 kJ/mol.^54^ We will revisit this in the context of the case study presented in the next section.

## 4 Case Study

We consider potential cocrystal formation for three APIs (see section 4.1) with 10 candidate coformers/solvents (see section 4.2). In all 30 cases, we performed parallel computational and experimental investigations at Imperial College London and University of Innsbruck, respectively.

A further CSP investigation was performed for the carbamazepine/aspirin cocrystal, for which an experimental structure (TAZRAO) was already reported in the CSD. While such a system would not normally be investigated within a coformer screen, it provides an additional data point on the ability of the approach to assess the likelihood of cocrystal formation.

### 4.1 APIs

The APIs used in this study are listed in Table 1. They were chosen as they are small, moderately flexible molecules with different tendencies toward cocrystal formation, and their crystal structures have been well studied in the literature.

**Table 1:** APIs Studied in This Case Study^a^

API	CSD reference code family	experimentally observed solid forms with resolved atomic coordinates
paracetamol	HXACAN	*Z*′ = 1: I, II
		*Z*′ = 2: III, VII
		*Z*′ = 4: VI
aspirin	ACSALA	*Z*′ = 1: I, II
		*Z*′ = 2: IV
carbamazepine	CBMZPN	*Z*′ = 1: II, III, IV, V
		*Z*′ = 4: I

a Forms IV and V of paracetamol and form III of aspirin are omitted as their atomic coordinates are not fully resolved.

**Figure 1:**
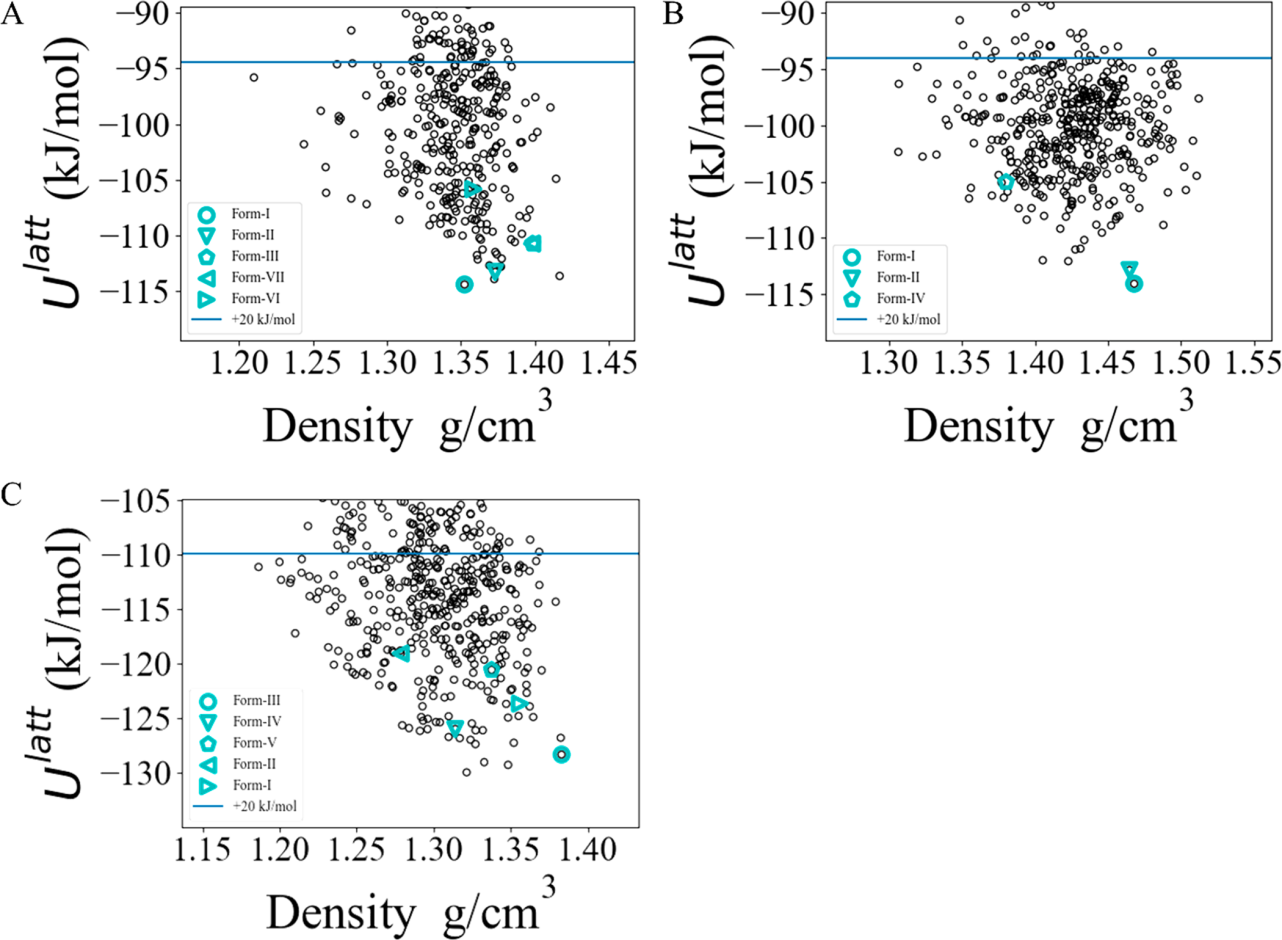
Single-component solid-form landscapes for the APIs (A) paracetamol, (B) aspirin, and (C) carbamazepine.

The CSP methodology outlined in section 2.1 was applied to each of the three APIs, in *Z*′ = 1, to create the corresponding solid-form landscapes shown in Figure 1. In the aspirin and paracetamol cases, the global lattice energy minima determined by the CSP are known experimental forms. In the carbamazepine case, the global minimum is 1.6 kJ/mol below the lowest-energy experimental form; all experimental forms were found within 11 kJ/mol of the global minimum. Furthermore, the geometries are well reproduced, with average RMSD_15_ values of 0.25 Å, suggesting that the “standard” CSP methodology detailed in section 2.1 is sufficient to describe systems of this size, flexibility, and elemental composition.

### 4.2 Candidate Coformers

The 10 candidate coformers considered in the investigation are listed in Table 2, as are the crystal structures reported in the CSD for them. They were selected from the GRAS list as molecules with moderate size and flexibility.

**Table 2:** Coformers Studied in This Paper

coformer	abbreviation	CSD reference code family
pyridoxine	BITZ	BITZAF
methyl paraben	CEBG	CEBGOF
3-*t*-butyl-4-hydroxyanisole	ESAL	ESALUF
propyl-4-hydroxybenzoate	DUPK	DUPKAB
nicotinic acid	NICC	NICOAC
nicotinamide	NICM	NICOAM
oxalic acid	OXAL	OXALAC
pyridine	PYRD	PYRDNA
succinic acid	SUCA	SUCACB
*cis*-aconitic acid	TELZ	TELZOZ

The CSP methodology outlined in section 2.1 was applied to each of the 10 coformers to create the lattice energy landscapes for the corresponding neat crystals. In 7 out of the 10 cases, the global minimum determined by the CSP was the known experimental form. For pyridoxine (BITZ), 3-*t*-butyl-4-hydroxyanisole (ESAL), and *cis*-aconitic acid (TELZ), the experimental forms are, respectively, 5.95, 2.92, and 1.72 kJ/mol above the corresponding global minima. The large error in the case of BITZ arises as a result of intramolecular hydrogen bonds being broken within the crystalline environment, an effect that is not well modeled using the isolated-molecule approximation;^55^ this is discussed in more detail in the Supporting Information. The geometries are well reproduced in all cases, with average RMSD_15_ values of 0.29 Å.

**Figure 2:**
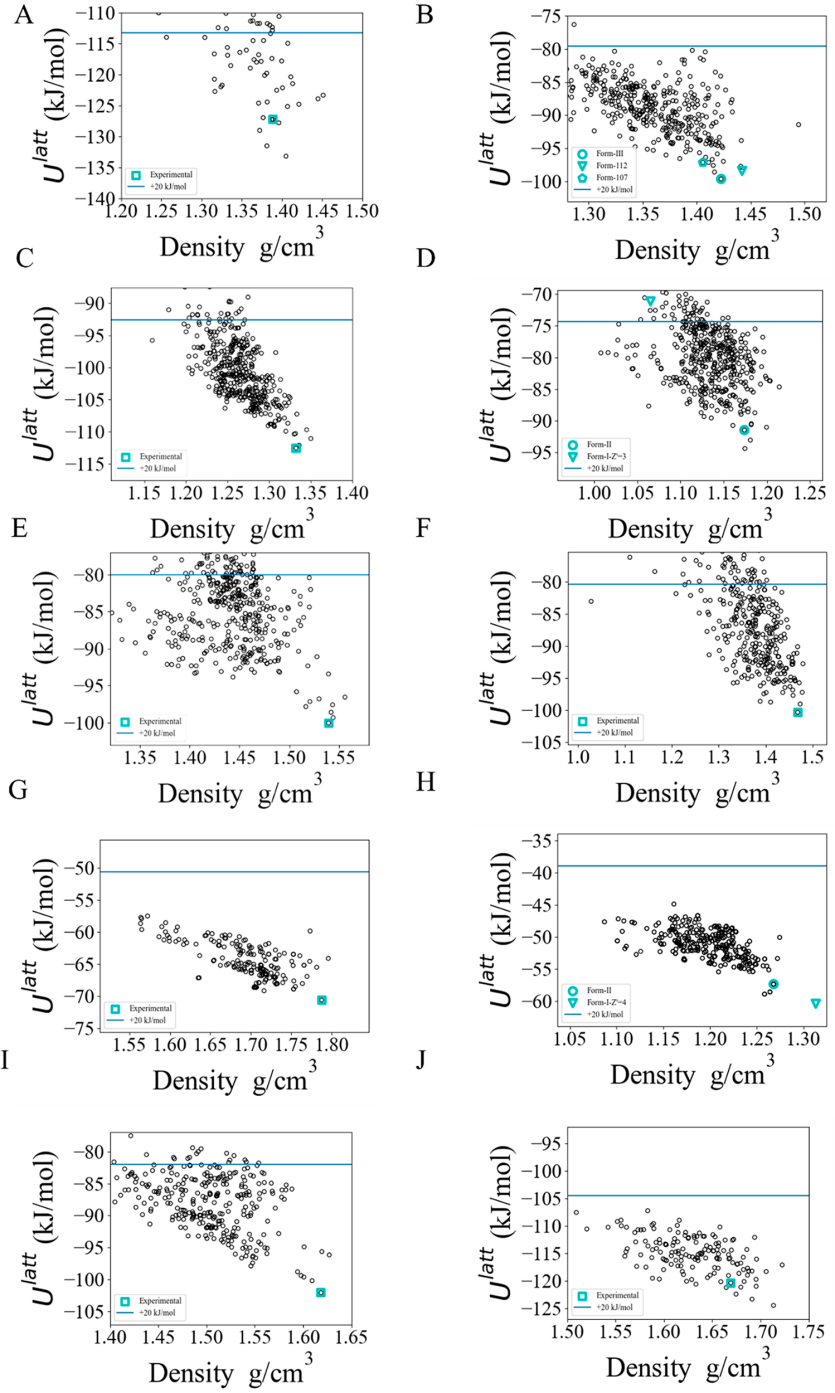
Single-component solid-form landscapes for the GRAS list coformers, (A) BITZ, (B) CEBG, (C) DUPK, (D) ESAL (*Z*′ = 2 landscape), (E) NICC, (F) NICM, (G) OXAL, (H) PYRD (*Z*′ = 4 experimental form included), (I) SUCA, and (J) TELZ. Blue horizontal lines indicate a 20 kJ/mol cutoff from the globally minimal lattice energy.

### 4.3 Experimental Investigations

The experimental search for cocrystals employed the range of techniques outlined in section 2.2 and successfully reproduced all known cocrystals and solvates; it also led to the identification of an additional five cocrystals of carbamazepine, as summarized in Table 3.

**Table 3:** Cocrystals and Solvates Identified via Experimental Screen

		cocrystal/solvate solid forms	
molecule #1	molecule #2	ref	known/new
paracetamol	oxalic acid	LUJTAM	known
paracetamol	pyridine	KUNTUK	known
carbamazepine	nicotinamide	UNEZES	known
carbamazepine	oxalic acid	MOXWUS	known
carbamazepine	succinic acid	XOBCIB	known
carbamazepine	methyl paraben	CARB/CEBG-A	new
carbamazepine	methyl paraben	CARB/CEBG-B	new
carbamazepine	3-*t*-butyl-4-hydroxyanisole	CARB/ESAL-A	new
carbamazepine	3-*t*-butyl-4-hydroxyanisole	CARB/ESAL-B	new
carbamazepine	*cis*-aconitic acid	CARB/TELZ	new

Liquid-assisted grinding and slurry experiments were identified as the most successful methods to find and isolate phase pure cocrystals. The contact preparation method and sublimation of the coformers lead only to two and one hits, respectively. The low hit rate can be related to the limitation of the methods. High melting point differences between API and coformer and the decomposition of certain coformers at the melting temperature limit the applicability of the contact preparation method. In case of cosublimation, vapor deposition of the two components should occur at similar temperatures, which was not the case for the majority of the chosen combinations. Solvent evaporation, a commonly used technique to produce single crystals of cocrystals, was only applied for certain combinations and therefore not contrasted to the hit rates of the other used methods (see sections 2.2–2.4 of the Supporting Information).

The PXRD from the cocrystallization experiments from carbamazepine and propyl 4-hydroxybenzoate (CARB/DUPK) indicates the potential existence of a cocrystal. However, as this was not fully conclusive, this cocrystal is not included in Table 3. A new polymorph of *cis-*aconitic acid (TELZ) (form II) was also identified during the experiments. For more details, see section 3.4 of the Supporting Information.

#### 4.3.1 CARB/ESAL Cocrystals (CARB/ESAL-A and CARB/ESAL-B)

**Figure 3:**
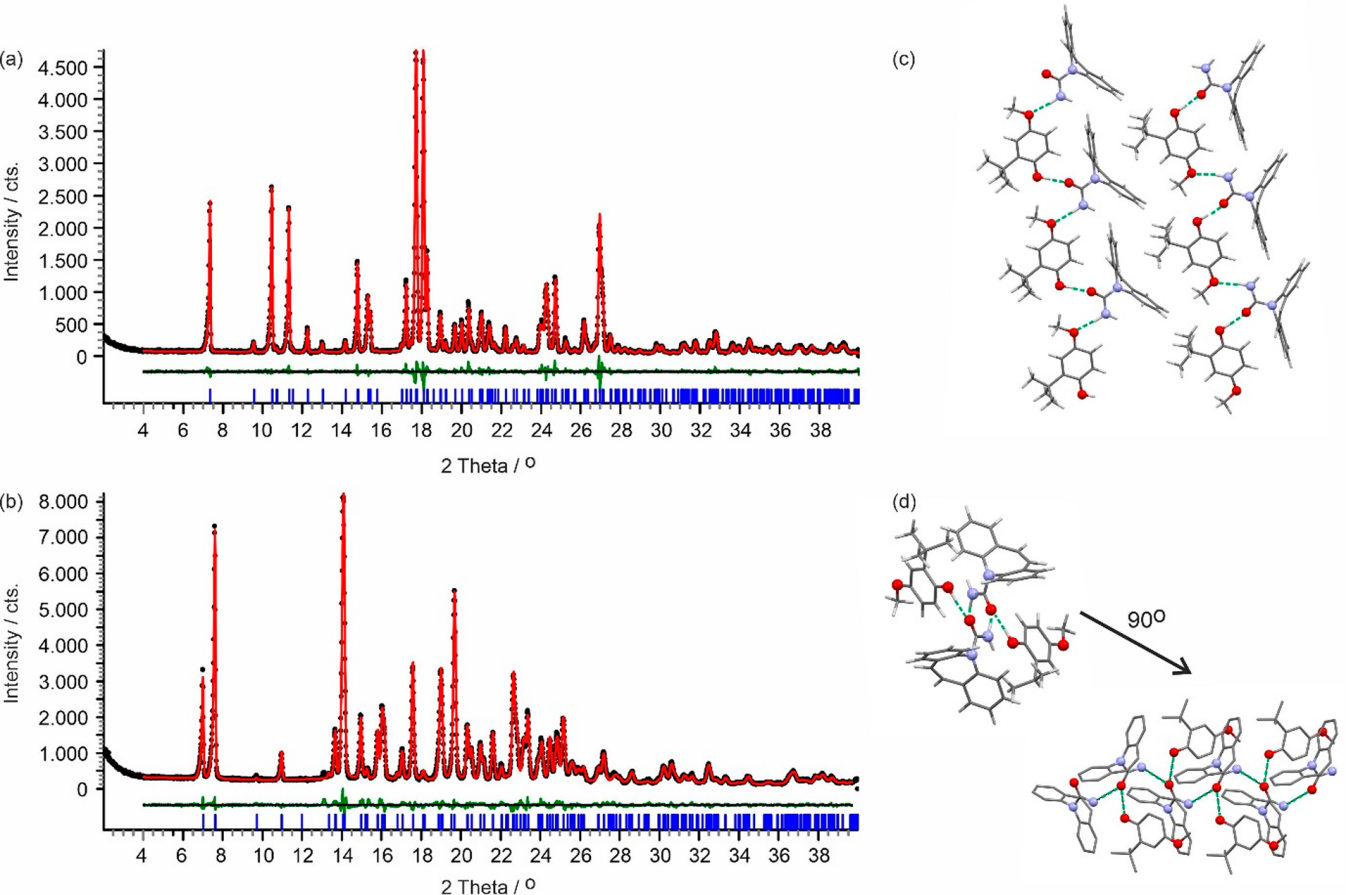
Observed (black points), calculated (red line), and difference profiles (green) for the Rietveld refinements of CARB/ESAL polymorphs A (a) and B (b). Blue tick marks denote the peak positions. Note that form B shows a phase impurity (CARB) at 13.1 and 15.3° 2θ. (c, d) Hydrogen bonding motifs showing one layer for form A (panel c) and the packing motif of the 2_1_-mediated carbamazepine chain and ESAL···CARB interactions for form B (panel d). Green (dotted) lines indicate the strong H-bonding interactions. Hydrogen atoms are omitted in panel (d) for clarity.

Polymorph CARB/ESAL-A was obtained with LAG experiments using *n*-heptane. It crystallized in the monoclinic space group *P*2_1_/*n* (Figure 3a) with one CARB and one ESAL molecule in the asymmetric unit, the conformation of the ESAL molecule being related to the ones seen in ESAL form I (ESALUF, *Z*′ = 3). Strong hydrogen bonding interactions are exclusively formed between the two components, one O–H···O and one N–H···O, leading to a C_2_^2^(11) chain propagating parallel to the *a* crystallographic axis (Figure 3c). Adjacent chain motifs form strong C–H···ring contacts, forming a layer-like arrangement in (0 0 4). Adjacent layers are related by inversion symmetry, resulting in the 3D packing of CARB/ESAL-A.

A second polymorph, CARB/ESAL-B, was obtained with LAG experiments using dichloromethane. It also adopts the monoclinic crystal symmetry, *P*2_1_/*c* and *Z*′ = 1 (Figure 3b). The carbamazepine molecules essentially adopt the same conformation, but the orientation of the methyl group of the ESAL molecule differs by approximately 180°. Thus, the conformation of the ESAL molecule can be related to the one seen on ESAL form I (ESALUF01, *Z*′ = 1). The carbamazepine molecules form N–H···O C_1_^1^(4) hydrogen bonding interactions, related by 2_1_ symmetry and propagating parallel to the *b* crystallographic axis. The ESAL molecule interacts through a strong O–H···O interaction with the carbamazepine (Figure 3d). Furthermore, the carbamazepine molecule forms C–H···ring contacts.

#### 4.3.2 CARB/CEBG Cocrystals (CARB/CEBG-A and CARB/CEBG-B)

**Figure 4:**
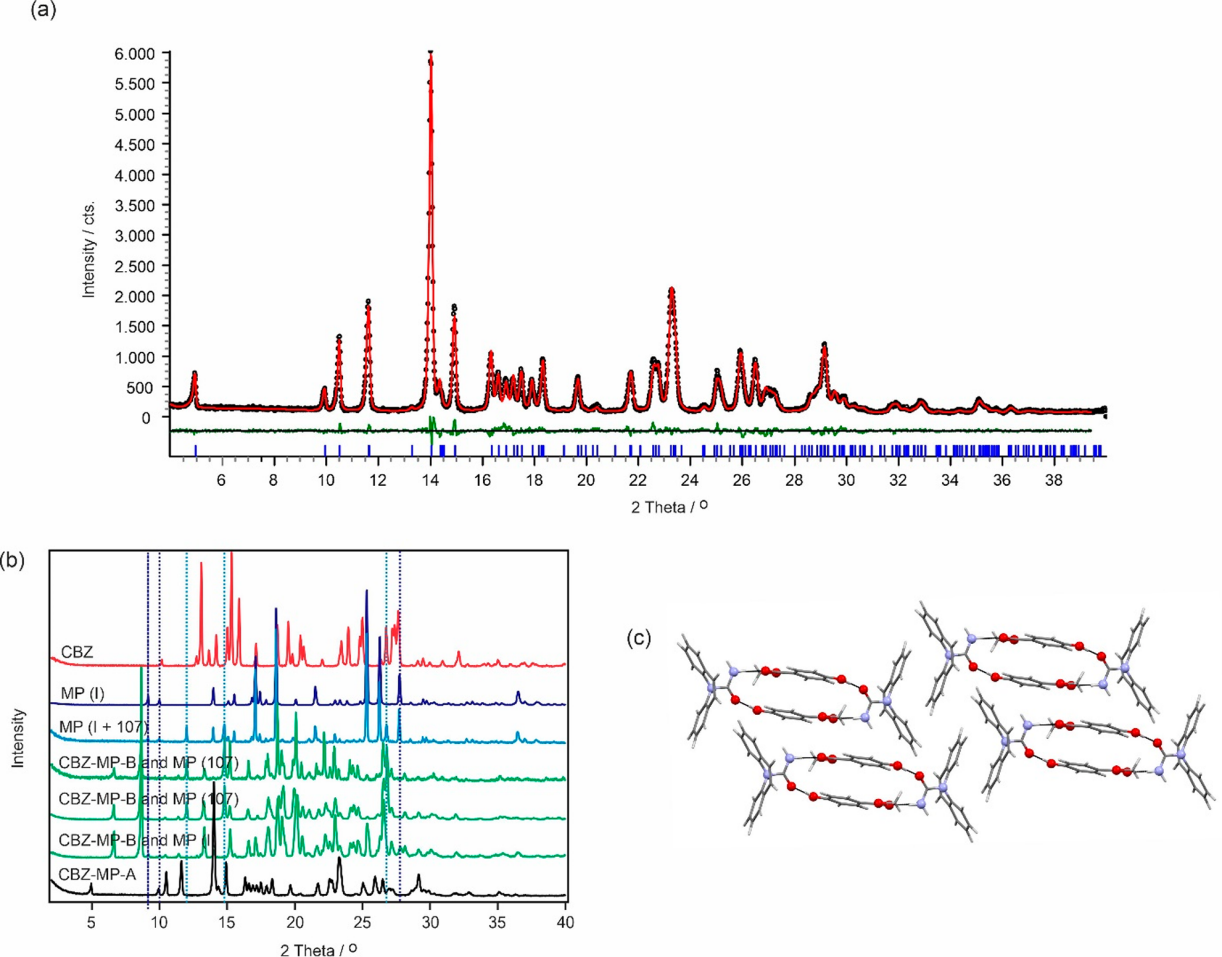
(a) Observed (black points), calculated (red line), and difference profiles (green) for the Rietveld refinements of the CARB/CEBG-A cocrystal. Blue tick marks denote the peak positions. (b) Comparison of the carbamazepine (CARB, red), methyl paraben (polymorph I and a mixture of I and 107, blue) products obtained after evaporating 1:1 mixtures of the two components from acetonitrile and ethanol (green), and the CARB/CEBG-A cocrystal (black). Dotted lines indicate selected key reflection peak positions of methyl paraben polymorphs I and 107. (c) Packing diagram CARB/CEBG-A viewed along the *a* crystallographic axis.

Recrystallization from the melt of a 1:1 mixture of the two components and slurry experiments (dichloromethane, diethyl ether, and *n*-heptane) led to a new cocrystal (Figure 4a), here denoted as CARB/CEBG-A. The latter crystallizes in the triclinic *P*1 ®symmetry (Figure 4a), with one carbamazepine and one methyl paraben molecule in the asymmetric unit, confirming the 1:1 stoichiometry. The carbamazepine molecule forms strong hydrogen bonding interactions to two methyl paraben molecules, involving the amide function of CARB (C=O and N–H) and ester =O and hydroxyl group of the coformer (Figure 4c). The two methyl paraben molecules are related by inversion symmetry and stabilize due to the aromatic ring stacking (π···π) the packing. The strong O–H···O and N–H···O hydrogen bonds form a tetrameric ring motif, R_4_^4^(24), with adjacent ring motifs being inversion symmetry related.

Evaporation from a room temperature saturated equimolar solution of the two components (from either acetone, acetonitrile, or ethanol) resulted concomitantly in a distinct cocrystal form (CARB/CEBG-B) and methyl paraben (Figure 4b). Depending on the solvent used, methyl paraben forms I and/or form 107^56^ were present. The presence of methyl paraben and the absence of carbamazepine, after complete evaporation of the solvent, indicates that the obtained cocrystal B exhibits a >1:1 stoichiometric ratio.

#### 4.3.3 CARB/TELZ Cocrystal

**Figure 5:**
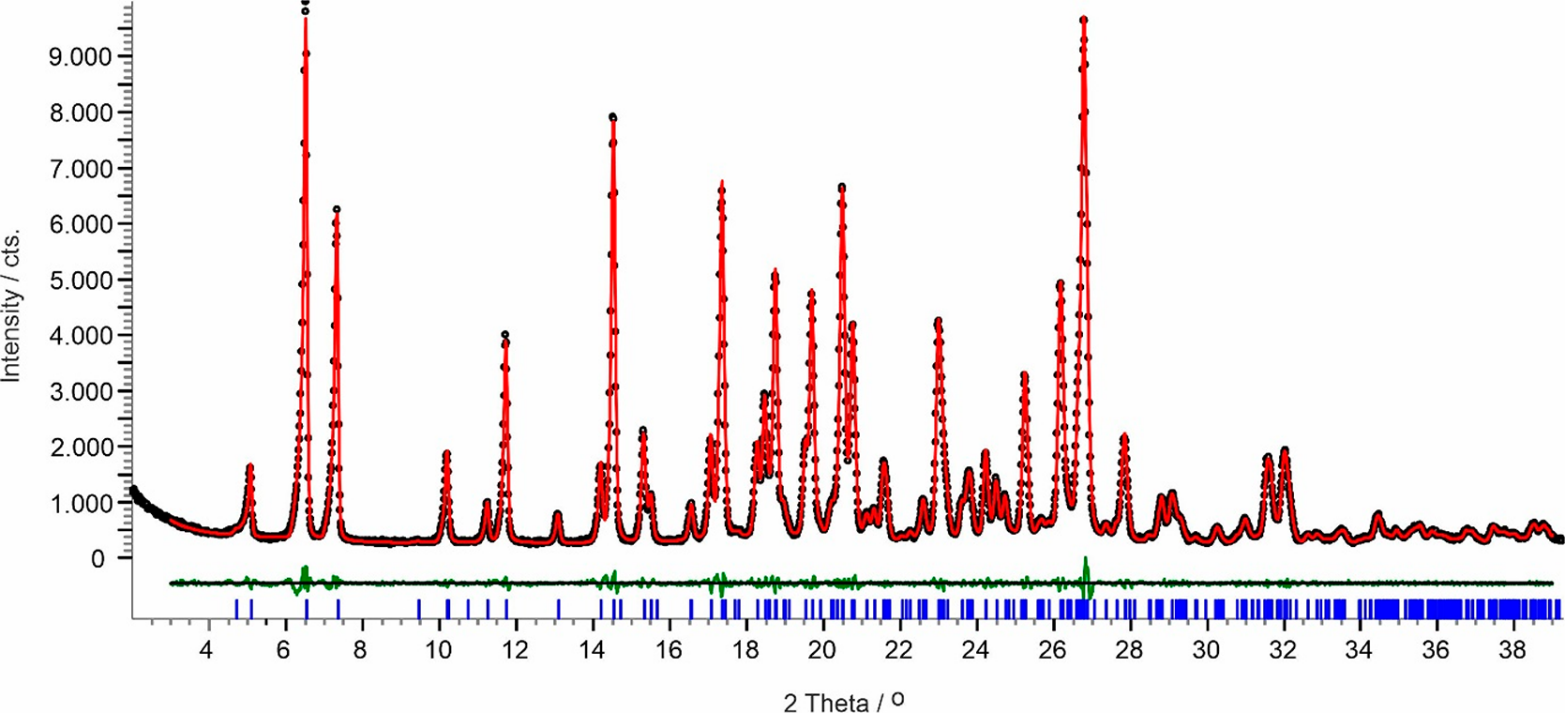
For the *hkl* reflections (*R*_wp_ = 3.91%) between the reflection PXRD data of CARB/TELZ with a model consisting of the cell parameters derived from indexing. Black dots indicate raw data, while the red line indicates the calculated model. The difference pattern is shown in green. Tick marks (blue) are the 2θ positions for the *hkl* reflections Pawley fit.

Cogrinding experiments, using *n*-heptane or diethyl ether as solvents, resulted in a new carbamazepine cocrystal with *cis*-aconitic acid (TELZ) and *cis*-aconitic acid impurities. Slurry experiments in the same solvents resulted in the cocrystal phase only. The following cell, indexed using DICVOL04 (see section 2.2) and a F(20) of 57.2, is the metric for the cocrystal: *P*2_1_/*n*, *a* = 27.0723(10) Å, *b* = 5.1939(2) Å, *c* = 24.1059(10) Å, β = 94.343(2)°, *V* = 3379.80(22) Å^3^ (Figure 5). On the basis of the cell volume, it is evident the cocrystal does not exhibit a 1:1 ratio but points to a 2:1 stoichiometry (CARB/TELZ).

### 4.4 Cocrystal CSP Investigations

The methodology outlined in section 3 was applied to screening the 10 candidate coformers as potential cocrystallizing agents for each of the three APIs. We limit our screening to 1:1 cocrystals API_1_.*c*_1_ (*m* = *n* = 1), and the neat-crystal lattice energies *U*_[API],min_ and *U*_[*c*],min_ used in eqs 2 and 5 are computed based on the most stable experimentally available forms (cf. sections 4.1 and 4.2).

**Figure 6:**
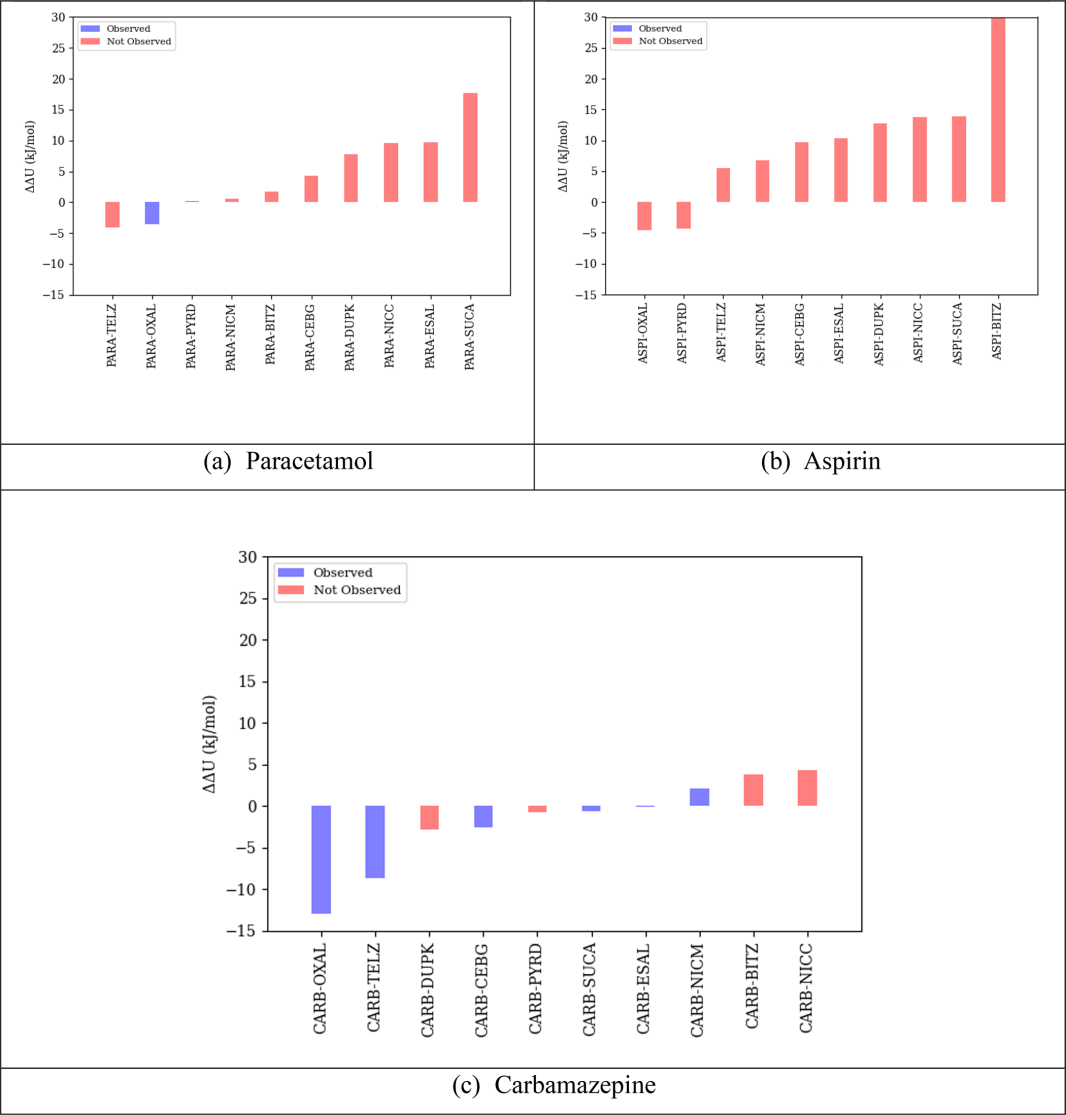
Ranked values of ΔΔ*U* for potential cocrystals of (a) paracetamol, (b) aspirin, and (c) carbamazepine. Blue bars indicate API/coformer pairs for which cocrystals have been observed experimentally. Neat-crystal lattice energies for API and coformers in eqs 2 and 5 were based on the most stable experimentally observed crystal structures.

The results of the algorithm of section 3 are shown in Figure 6 in terms of the ranked values of the lattice energy difference quantity ΔΔ*U* for the 10 candidate coformers and given in more detail in section 1.3 of the Supporting Information. It is noted that, since pyridine is liquid at room temperature (*T*_fus_ = 231.5 K; Δ*H*_fus_ = 8.3 kJ/mol), eq 5 is used for all pyridine cocrystals; this effectively increases the value of ΔΔ*U* by 2.5 kJ/mol. Equation 2 was used for computing quantity ΔΔ*U* for all other cocrystals.

The blue bars indicate API–coformer pairs for which cocrystals have actually been observed experimentally, albeit not necessarily in the 1:1 stoichiometry. As mentioned in section 4.3, the experimental results also indicate that the carbamazepine/propyl 4-hydroxybenzoate (CARB–DUPK) pair may also have resulted in a cocrystal. Although this was not conclusive, as we were not able to grow suitable single crystals or solve the structure from PXRD, the existence of that cocrystal would seem to be corroborated by the negative value of the corresponding ΔΔ*U*.

**Figure 7:**
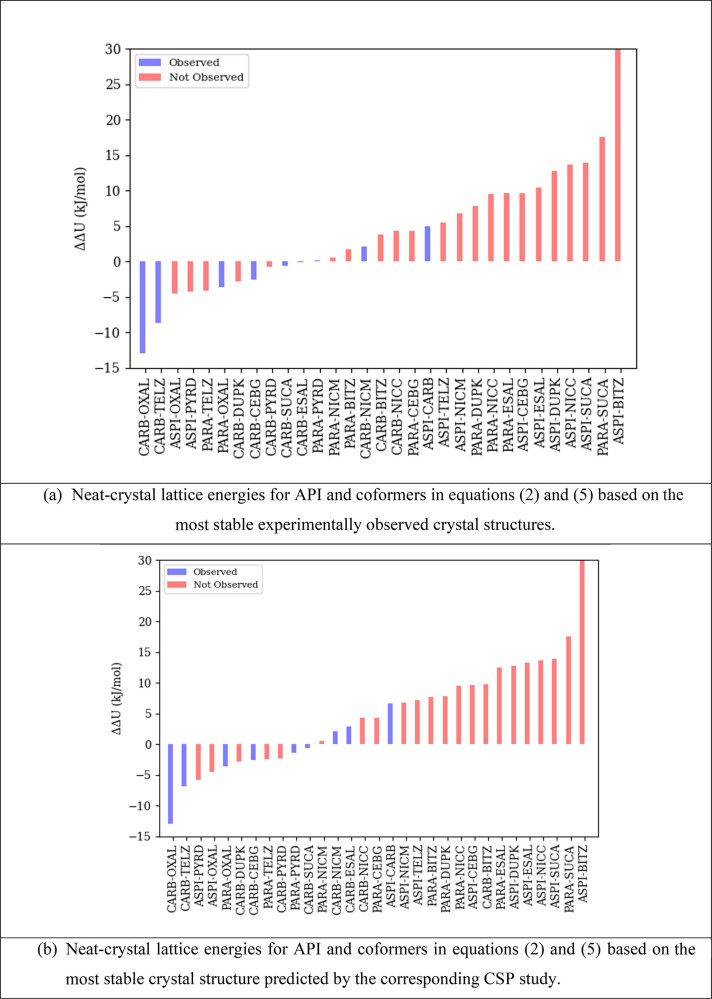
(a, b) Combined ranked values of ΔΔ*U* for potential cocrystals of paracetamol, aspirin, and carbamazepine, including the aspirin/carbamazepine (ASPI/CARB) cocrystal. Blue bars indicate API/coformer pairs for which cocrystals have been observed experimentally.

The combined results for all three APIs are shown in Figure 7a. The latter also includes the known aspirin/carbamazepine cocrystal (TAZRAO, ASPI/CARB). TAZRAO is especially interesting as it combines an API that readily forms cocrystals (carbamazepine, with 6 identified cocrystals out of the 10 potential coformers) and one that does not (aspirin, with no identified cocrystals).

As can be seen, among the 10 coformers, there is a strong correlation between a low value of ΔΔ*U* and the realization of the experimental cocrystal. All the cocrystals that are observed experimentally are found within a ΔΔ*U* value of 5 kJ/mol, with the highest value corresponding to the aspirin/carbamazepine pair. This pair is ranked 11th among all carbamazepine pairs but only third among all aspirin pairs; this is consistent with the cocrystallization propensity of these two compounds.

Overall, the results indicate that, although a very negative value of ΔΔ*U* for a given potential cocrystal does not guarantee its existence, a value exceeding 5 kJ/mol makes it unlikely. Therefore, an experimental coformer screening program may exclude any coformer *c* with ΔΔ*U*_*c*_ > 5 kJ/mol. As indicated by the statistics presented in Table 4, this could significantly increase both efficiency (by avoiding investigation of coformers that are unlikely to lead to cocrystals) and effectiveness (by improving the proportion of investigations that are successful).

**Table 4:** Potential Effects of Computational Coformer Screening on Experimental Coformer Screening Programs^a^

API	% coformers with ΔΔ*U*_*c*_ > 5 kJ/mol	% coformers with ΔΔ*U*_*c*_ ≤ 5 kJ/mol that lead to experimentally observed cocrystals
paracetamol	40	33
aspirin	80	0
carbamazepine	0	60
**overall**	**40**	**44**

a The second column indicates the proportion of the coformers that could be deemed to be unlikely to lead to cocrystals and might therefore be excluded from an experimental program. The last column indicates the proportion of nonexcluded coformers that would lead to a successful identification of a cocrystal.

As shown in Figure 7a, six out of the nine cocrystals that have been observed experimentally have negative ΔΔ*U*_*c*_ values, which would indicate that, based on a lattice energy measure of stability, the cocrystal is more stable than its constituents. Therefore, one might consider using a criterion of ΔΔ*U*_*c*_ ≤ 0 to decide whether to perform an experimental investigation involving a particular coformer *c*. This would lead to further improvements to both the efficiency and effectiveness of the experimental screening program: for the three APIs considered here, 19 of the 30 potential investigations would be eliminated; and 6 of the 11 investigations that would be performed would lead to the successful identification of a cocrystal. However, as indicated by the results presented here, these improvements come at the risk of missing an existing cocrystal. This may be the result of deficiencies in the underlying CSP studies, such as inaccuracies in the lattice energy model, incomplete search for lower-energy structures, consideration of only specific stoichiometries, and the failure to take account of entropic effects. More importantly, it is worth bearing in mind that a cocrystal may be observed experimentally even if it is metastable with respect to its constituents.^54,57^ This is consistent with the situation with single-component crystals: using a comparable model of lattice energy to ours, Nyman and Day^58^ reported that 95% of observed lattice energy differences between neat polymorphs of the same molecule are within 7.2 kJ/mol. Overall, time and resources permitting, it would appear to be prudent for experimental screening programs to include coformers with positive ΔΔ*U*_*c*_ values up to +5 kJ/mol.

The results presented in Figure 6 and Figure 7a were obtained with the quantities *U*_[API],min_ and *U*_[*c*],min_ in eqs 2 and 5 being computed for the most stable experimentally observed crystal structures for the API and the coformers, respectively. Figure 7b presents results in which these quantities correspond to the most stable structures predicted by the respective CSP studies (cf. sections 4.1 and 4.2). A comparison with Figure 7a indicates that the results are broadly the same, with a strong correlation between experimental cocrystal formation and ΔΔ*U* values. However, the aspirin/carbamazepine cocrystal (TAZRAO) has a ΔΔ*U* above +5 kJ/mol. Overall, in deciding the scope of an experimental screening program, a higher cutoff might be appropriate in recognition of the increased uncertainty that arises from relying solely on computational data.

### 4.5 Computational Cost

The computational costs of the neat API and cocrystal investigations are summarized in Table 5. The times quoted do not include the cost of performing the neat coformer investigations, as that is a one-off calculation that would not need to be repeated for any new API. It is interesting to note that, as a result of the reuse of LAM databases, the cost of cocrystal CSP investigations is comparable to, or even lower than, the cost of the neat API investigations, despite the fact that the characterization of cocrystal unit cells involves many more variables.

**Table 5:** Computational Cost (in CPU Hours) of CSP Investigations^a^

	LAM generation for global search	global search	low-energy structure refinement	total
paracetamol				
neat	1086	827	4133	6057
cocrystal (per coformer)	N/A	1863	1591	3470
aspirin				
neat	3837	463	2763	7078
cocrystal (per coformer)	0.00	1827	3304	5134
carbamazepene				
neat	260	729	4342	5114
cocrystal (per coformer)	N/A	2447	3154	5629

a AMD EPYC 7742 processors were used for the global search, and AMD EPYC 7742 64-Core Processors or Intel(R) Xeon(R) CPU E5-2620 @ 2.00 GHz were used for LAM generation and refinement. Times quotes for cocrystals are averaged over all coformers studied. Totals include time required for analysis and clustering of structures between global search and refinement stages.

Note that all of the computational steps are highly parallelizable: the QM calculations for generating LAMs at different molecular conformations prior to the global search may be performed in parallel; the global search involves the generation of hundreds of thousands of candidate crystal structures and their subsequent lattice energy minimization, and these can also be carried out in parallel; the final refinement of hundreds of low-energy crystal structures can also be parallelized. The CrystalPredictor and CrystalOptimizer codes used for the CSP studies fully exploit this potential for parallelizability. Additionally, for any given API, the CSP studies for cocrystals using different coformers can also be performed in parallel if sufficient computational resources are available.

In our calculations, we employed computational clusters comprising 32 cores for the LAM generation and refinement calculations, and 512 cores for the global search. This allowed each CSP study for a neat API to be completed within about 3–5 days of wall time. The CSP study for each API/coformer cocrystal took 2–3 days of wall time. Moreover, given sufficient computational resources, multiple potential cocrystals for the same API could be investigated in parallel. Overall, these statistics indicate that computational screening could provide useful input to drug product development on an acceptable time scale.

## 5 Concluding Remarks

This paper has presented a computational procedure for cocrystal screening for a given active pharmaceutical ingredient (API) against a set of potential coformers. The assessment of the likelihood of formation of a stable cocrystal is based on the difference ΔΔ*U* between the lattice energy of the most stable cocrystal and the sum of the lattice energies of the most stable neat forms of its constituents. An *ab initio* crystal structure prediction (CSP) methodology is used to determine the most stable cocrystal form. For the API and coformers, the most stable form may either be determined in the same manner or already be known experimentally.

A key characteristic of the proposed approach is its computational efficiency. This is achieved by eliminating the need for any isolated-molecule quantum mechanical calculations beyond those already required for the neat API and neat coformer CSP studies. Overall, this allows the efficient screening of relatively large numbers of coformers.

An investigation involving the application of the proposed computational methodology to three different APIs, each screened against 10 potential coformers was carried out. This was complemented by a parallel experimental investigation on the same systems, aiming to identify as many cocrystals as possible using a variety of experimental techniques. A comparison of the results of the two investigations indicates that API/coformer pairs with a value of ΔΔ*U* exceeding 5 kJ/mol are unlikely to form cocrystals that can be obtained experimentally. This provides a useful criterion based on which unnecessary experimental investigations can be avoided, thereby resulting in significant savings in both time and API material. Future plans will focus on using the technique on larger and more flexible APIs that are of more relevance to modern pharmaceutical developmental workflows.

## Supporting Information Available

A breakdown of the computational costs and the values used to calculate ΔΔ*U*_c_; Rietveld refinements and, where appropriate, packing diagrams, for all of the new forms discovered in the experimental search; overviews of the experimental results, broken down by experiment, including experimental and simulated PXRD diagrams for each API, coformer, and API–coformer pair; images of the formation of the CARB–NICM cocrystal in the contact preparation method; details of the extra experimental efforts made for the CARB–DUPK pair; Rietveld refinement methodology, Shelx format crystal structure files, and packing similarity images between calculated and simulated structures for each of the newly discovered forms (PDF)
