# Supplementary material for: Efficient Screening of Coformers for Active Pharmaceutical Ingredient Cocrystallization
Source: Cryst Growth Des. 2022 Jun 15;22(7):4513–27. doi: 10.1021/acs.cgd.2c00433 (PMC9337750; doi:10.1021/acs.cgd.2c00433)
Supplement: Supplementary file 1 — cg2c00433_si_001.pdf [file cg2c00433_si_001.pdf]

Supporting Information: Efficient Screening of Coformers for Active Pharmaceutical Ingredient Cocrystallization

Isaac J. Sugden*[,1,#] Doris E. Braun*[,2,#] David H. Bowskill,[1] Claire S. Adjiman[1] and Constantinos C. Pantelides[1]

1. Molecular Systems Engineering Group, Department of Chemical Engineering, Centre for Process Systems Engineering, Institute for Molecular Science and Engineering, Imperial College London London SW7 2AZ, United Kingdom

2. University of Innsbruck, Institute of Pharmacy, Pharmaceutical Technology, Josef-Moeller-Haus, Innrain 52c, A-6020, Innsbruck, Austria

*Co-corresponding authors.
[#]These two authors contributed equally.

1 CPU cost breakdown

1.1 Single component investigations

Breakdown of CPU cost (CPU hours)

API	CrystPred LAMs	Global	Analysis	Clustering	CrystOpt refinements	Total
PARACETAMOL	1086.05	827.23	9.77	1.10	4132.59	6056.75
ASPIRIN	3837.39	463.20	10.79	3.80	2763.00	7078.18
CARBAMAZAPENE	37.22	729.36	4.86	1.00	4342.00	5114.45

1.2 Cocrystal investigations

structure	Global	Analysis	Clustering	refinements	refinement	Total
		**Aspirin**				
OXALAC	804	2	1	472	1717	2524
DUPKAB	4854	0	3	492	5445	10302
SUCACB	1129	0	0	497	2595	3724
CEBGOF	868	0	0	492	3444	4312
PYRDNA	954	2	1	499	1780	2737
TELZOZ	1292	0	0	498	3852	5144
NICOAM	1246	1	1	490	4075	5323
BITZAF	3130	0	3	491	1568	4701
NICOAC	1673	0	0	499	5556	7230
ESALUF	2317	17	2	495	3010	5347
		**Carbamazepine**				

OXALAC	1020	72	0	500	3221	4313
DUPKAB	3871	6	40	471	3306	7224
SUCACB	1644	18	0	266	1417	3079
CEBGOF	2395	55	5	499	3881	6335
PYRDNA	829	2	0	401	2851	3682
TELZOZ	958	0	3	500	3860	4822
NICOAM	1644	0	1	363	1364	3008
BITZAF	3596	1	7	308	5101	8706
NICOAC	1580	0	14	499	2063	3657
ESALUF	3384	57	14	499	3996	7451
ASPIRIN	5995	0	13	328	3630	9639
		**Paracetamol**				
OXALAC	1005	1	19	34	96	1120
DUPKAB	3708	1	7	483	2870	6585
SUCACB	868	2	31	499	1647	2548
CEBGOF	1221	1	10	415	1638	2870
PYRDNA	2297	23	0	197	915	3235
TELZOZ	1056	0	5	499	2753	3815
NICOAM	791	3	15	475	3267	4076
BITZAF	5249	3	0	204	978	6230
NICOAC	789	4	27	500	1739	2559
ESALUF	1647	0	3	497	9	1659

1.3 Computing quantity $\Delta\Delta U_c$

Table S3: single crystal CSP results

System	lowest experimental energy (kJ/mol)	Global Minimum (kJ/mol)
PARA	-114.405	-114.405
ASPI	-114.058	-114.058
CARB	-128.32	-129.947
BITZ	-127.253	-133.198
CEGB	-99.6216	-99.6216
DUPK	-112.607	-112.607
ESAL	-91.448	-94.3638
NICC	-100.044	-100.044
NICM	-100.317	-100.317
OXAL	-70.5857	-70.5857
PYRD	-60.4391	-58.9266
SUCA	-102.046	-102.046
TELZ	-114.033	-115.753

Table S4: Cocrystal CSP results and $\Delta\Delta U_c$ calculation with either scheme (all values in kJ/mol)

System	API exptal energy +coformer exptal energy	API exptal energy + coformer	Cocrystal energy	solvate correction	$\Delta\Delta U_c$ (approach 1)	$\Delta\Delta U_c$ (approach 2)

		GM energy				
PARA-BITZ	-241.659	-247.604	-239.915	0	1.7434	7.6887
PARA-CEBG	-214.027	-214.027	-209.705	0	4.3225	4.3225
PARA-DUPK	-227.012	-227.012	-219.217	0	7.79575	7.79575
PARA-ESAL	-205.853	-208.769	-196.186	0	9.6675	12.5833
PARA-NICC	-214.45	-214.45	-204.907	0	9.5424	9.5424
PARA-NICM	-214.723	-214.723	-214.198	0	0.5248	0.5248
PARA-OXAL	-184.991	-184.991	-188.554	0	-3.5631	-3.5631
PARA-PYRD	-171.774	-173.332	-177.185	2.5	-2.9104	-1.3525
PARA-SUCA	-216.452	-216.452	-198.819	0	17.6324	17.6324
PARA-TELZ	-228.438	-230.158	-232.549	0	-4.1106	-2.3908
ASPI-BITZ	-241.311	-247.256	-203.893	0	37.4178	43.3631
ASPI-CEBG	-213.68	-213.68	-203.994	0	9.686	9.686
ASPI-DUPK	-226.665	-226.665	-213.887	0	12.77795	12.77795
ASPI-ESAL	-205.506	-208.422	-195.11	0	10.3959	13.3117
ASPI-NICC	-214.102	-214.102	-200.377	0	13.725	13.725
ASPI-NICM	-214.375	-214.375	-207.614	0	6.7613	6.7613
ASPI-OXAL	-184.644	-184.644	-189.181	0	-4.5378	-4.5378
ASPI-PYRD	-171.427	-172.985	-181.3	2.5	-7.3737	-5.8158
ASPI-SUCA	-216.104	-216.104	-202.182	0	13.9226	13.9226
ASPI-TELZ	-228.091	-229.811	-222.617	0	5.4738	7.1936
CARB-BITZ	-255.573	-261.518	-251.738	0	3.8348	9.7801
CARB-CEBG	-227.941	-227.941	-230.526	0	-2.5843	-2.5843
CARB-DUPK	-240.927	-240.927	-243.73	0	-2.80365	-2.80365
CARB-ESAL	-219.768	-222.684	-219.803	0	-0.035	2.8808

CARB-NICC	-228.364	-228.364	-224.067	0	4.297	4.297
CARB-NICM	-228.637	-228.637	-226.569	0	2.06774	2.06774
CARB-OXAL	-198.905	-198.905	-211.832	0	-12.9269	-12.9269
CARB-PYRD	-185.688	-187.246	-192.044	2.5	-3.8559	-2.298
CARB-SUCA	-230.366	-230.366	-230.978	0	-0.612	-0.612
CARB-TELZ	-242.353	-244.073	-250.954	0	-8.6016	-6.8818
CARB-ASPI	-242.378	-244.005	-237.386	0	4.9916	6.6193

2 EXPERIMENTAL

2.1 *cis* Aconitic acid (form II)

Slurry experiments of the commercial sample in *n*-heptane, dichloromethane or diethyl ether resulted in form II. The experimental PXRD pattern of the *cis* aconitic acid form II indexed to the monoclinic space group *C*2/*c*, with Z'=1 (**Figure 1**.a). The unit cell and space group symmetry are distinct from the already known structure of *cis* aconitic acid form I (*Pbca*, Z'=1). The molecular conformations present in the two *cis* aconitic acid polymorphs differ substantially. In form I one of the acid groups forms an intramolecular O─H···O hydrogen bonding interaction to a second carboxylic acid function (**Figure 1**.b). This is in contrast to form II, where all three of the carboxylic acid protons form intermolecular interactions. Five strong hydrogen bonding interactions are formed in form I, one carboxylic acid dimer [(R_2^2(8)] [1], one C_1^1(7) chain and the intramolecular hydrogen bond. The second polymorph forms six hydrogen bonding interactions, two R_2^2(8) dimers one C_1^1(7) chain motif with all interactions being O─H···O (**Figure 1**.c). Furthermore, C─H···O close contacts stabilise the structure.

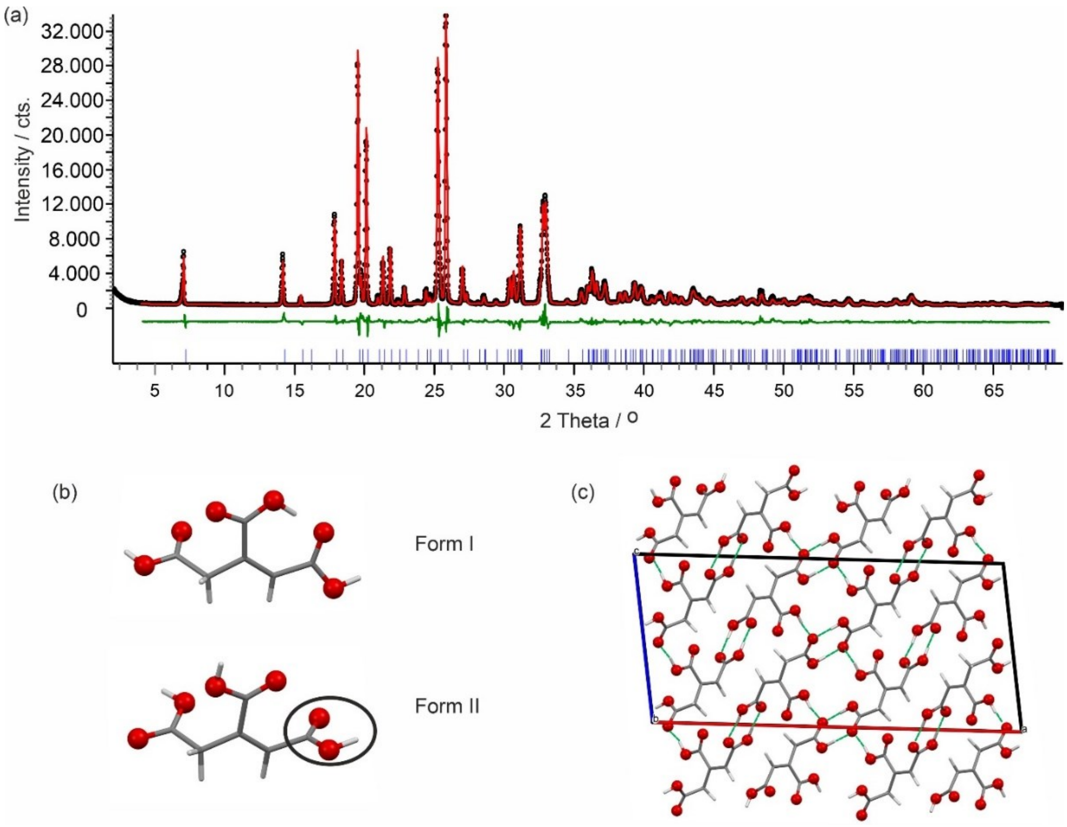

Figure 1. (a) Observed (black points), calculated (red line) and difference profiles (green) for the Rietveld refinements of *cis* aconitic acid form II. Blue tick marks denote the peak positions. (b) Conformation found in the two *cis* aconitic acid polymorphs. Note one of the COOH function of form II might be disordered, i.e. 180° flip of the acid function marked with an ellipsoid. (c) Packing diagram of *cis* aconitic acid form II viewed along the *b* crystallographic axis.

Finally, the *cis* aconitic acid pure form investigation is slightly different. Experimental indications suggested that the intramolecular hydrogen bond may be broken, necessitating broadening the search ranges, as the initial investigation had assumed the intramolecular hydrogen bond was maintained.

2.2 Paracetamol (PARA) cocrystal screen

The **oxalic acid cocrystal** (PARA-OXAL) and **pyridine solvate** (PARA-PYRD) were both successfully reproduced.

An overview over the paracetamol crystallization experiments is given in Table S1 and selected PXRD diffractograms are shown in Figure S1 - Figure S10.

Table S1. Overview **paracetamol** crystallization results.

Coformer	Contact Preparation	Slurry experiments			Liquid-assisted grinding		Co-sublimation
		n-Heptane	Pyridine	Diethyl ether	*n*-heptane	Diethyl ether	

Pyridoxine	x	x	n. a.	x	x	x	x
Methyl parabene	x	x	n. a.	x	x	x	x
Propyl parabene	x	x	n. a.	x	x	x	x
t-Butyl-4-hydroxyanisole	x	x	n. a.	x	x	x	x
Nicotinic Acid	n. a.	x	n. a.	x	x	x	x
Nictoinamide	x	x	n. a.	x	x	x	x
Oxalic Acid	n. a.	yes	n. a.	yes	yes	yes	yes
Succinic Acid	x	x	n. a.	x	x	x	x
cis-Aconitic Acid	n. a.	x	n. a.	x	x	x	x
Pyridine	n. a.	n. a.	yes	n. a.	n. a.	n. a.	n. a.

n.a. – not attempted, yes – cocrystal/solvate formation, x – physical mixture of the two compounds.

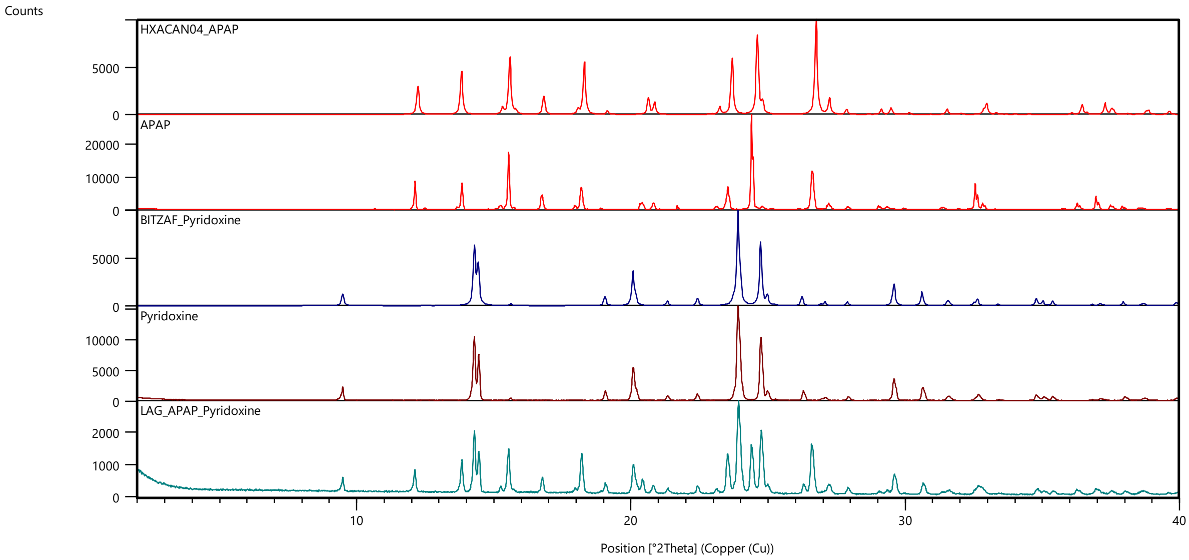

Figure S1. Comparison of experimental and from single crystal structure data simulated PXRD patterns of paracetamol (red), **pyridoxine** and a physical mixture obtained from LAG grinding experiments.

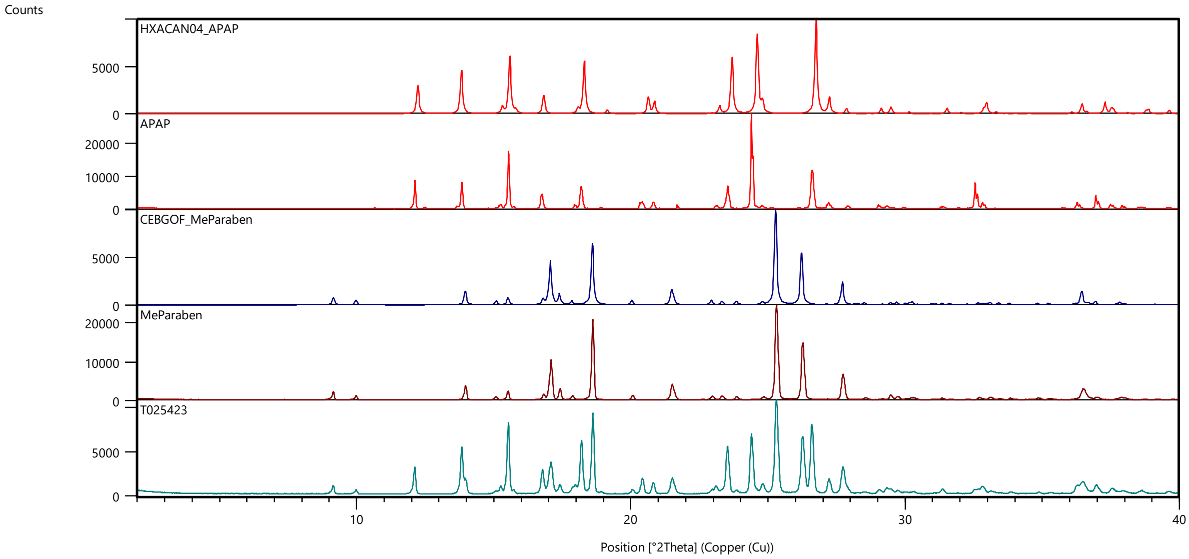

Figure S2. Comparison of experimental and from single crystal structure data simulated PXRD patterns of paracetamol (red), **methyl paraben** and a physical mixture obtained from LAG grinding experiments.

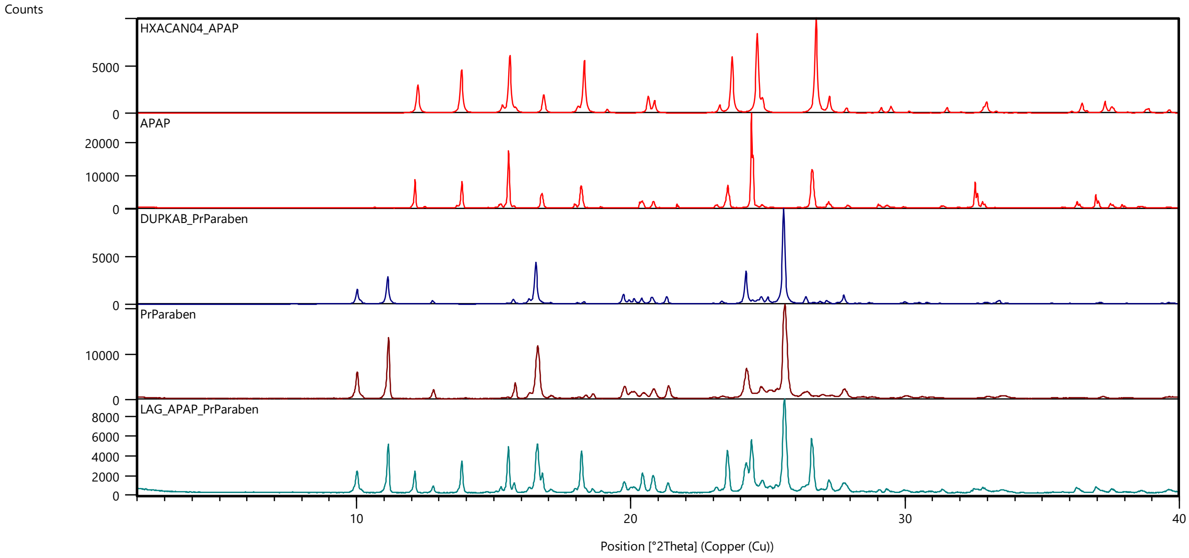

Figure S3. Comparison of experimental and from single crystal structure data simulated PXRD patterns of paracetamol (red), **propyl paraben** and a physical mixture obtained from LAG grinding experiments.

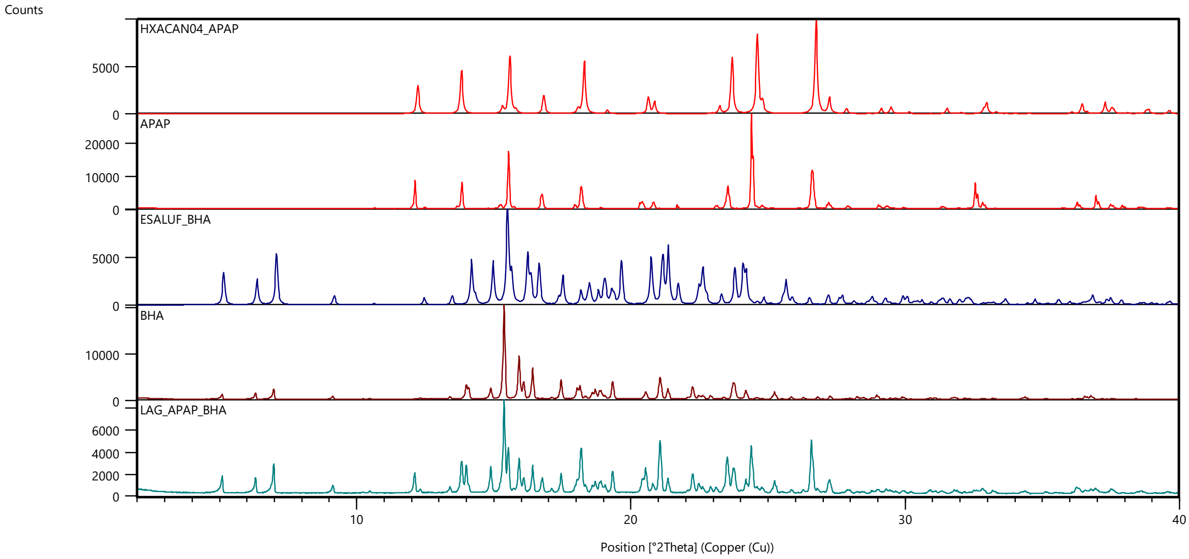

Figure S4. Comparison of experimental and from single crystal structure data simulated PXRD patterns of paracetamol (red), **t-butyl-4-hydroxyanisole** and a physical mixture obtained from LAG grinding experiments.

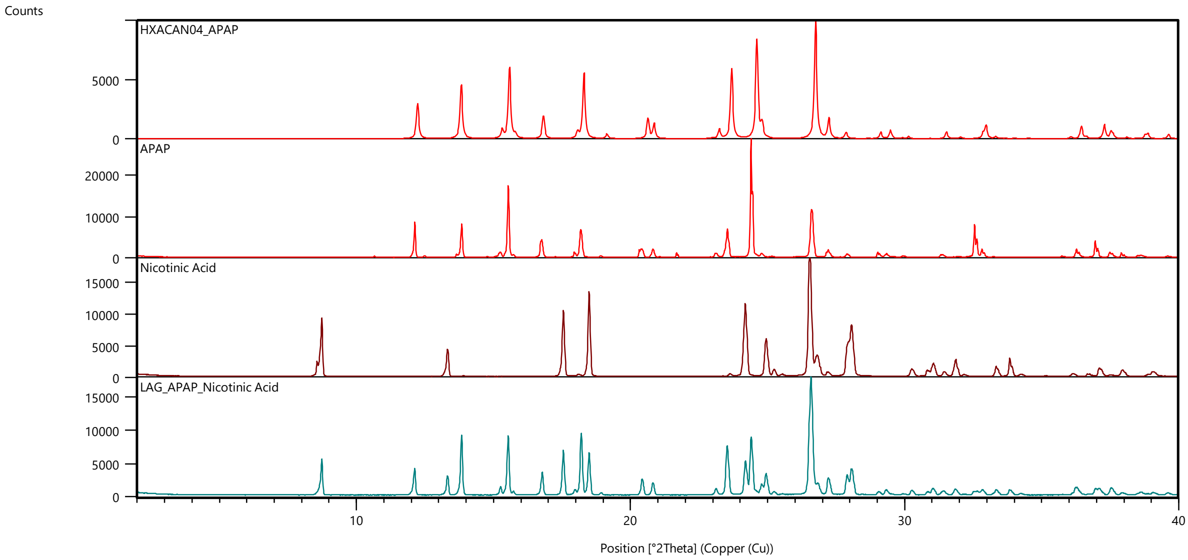

Figure S5. Comparison of experimental and from single crystal structure data simulated PXRD patterns of paracetamol (red), **nicotinic acid** and a physical mixture obtained from LAG grinding experiments.

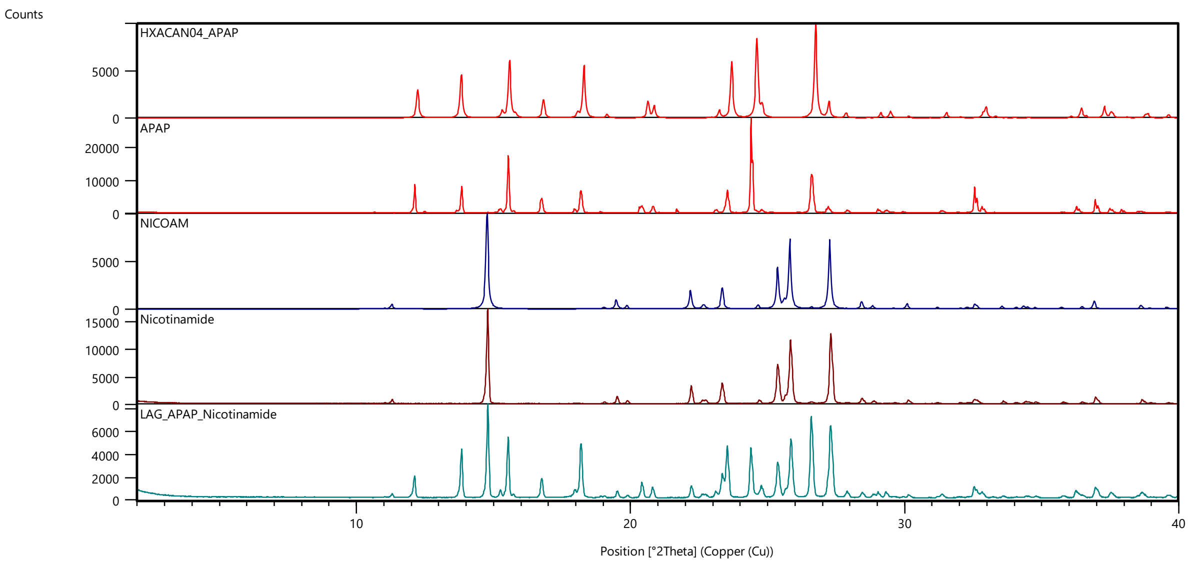

Figure S6. Comparison of experimental and from single crystal structure data simulated PXRD patterns of paracetamol (red), **nicotinamide** and a physical mixture obtained from LAG grinding experiments.

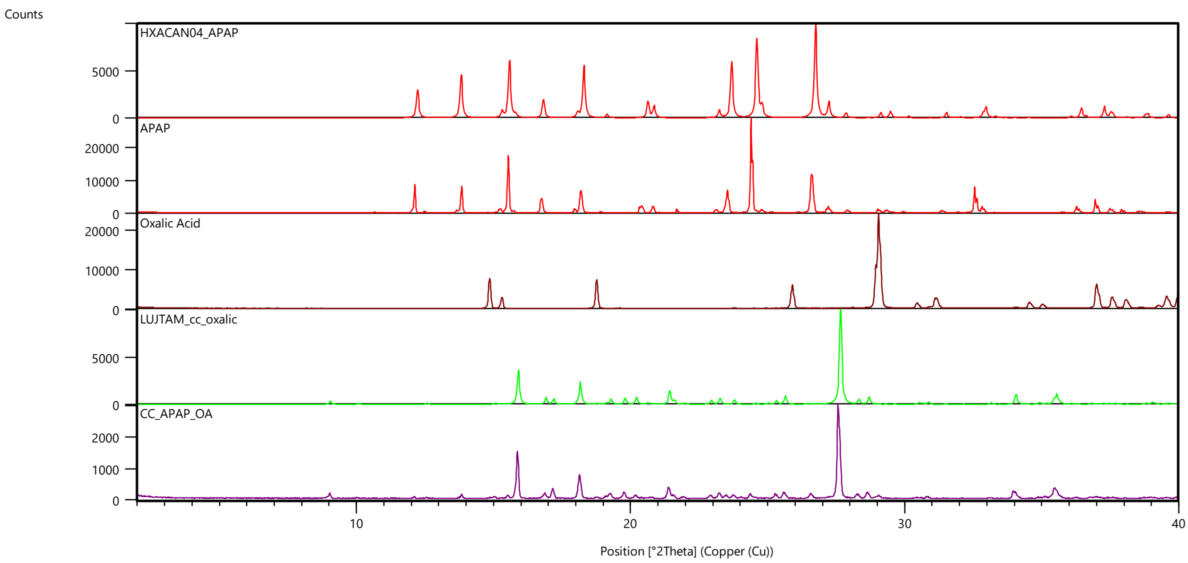

Figure S7. Comparison of experimental and from single crystal structure data simulated PXRD patterns of paracetamol (red), **oxalic acid** and the cocrystal obtained in co-sublimation experiments.

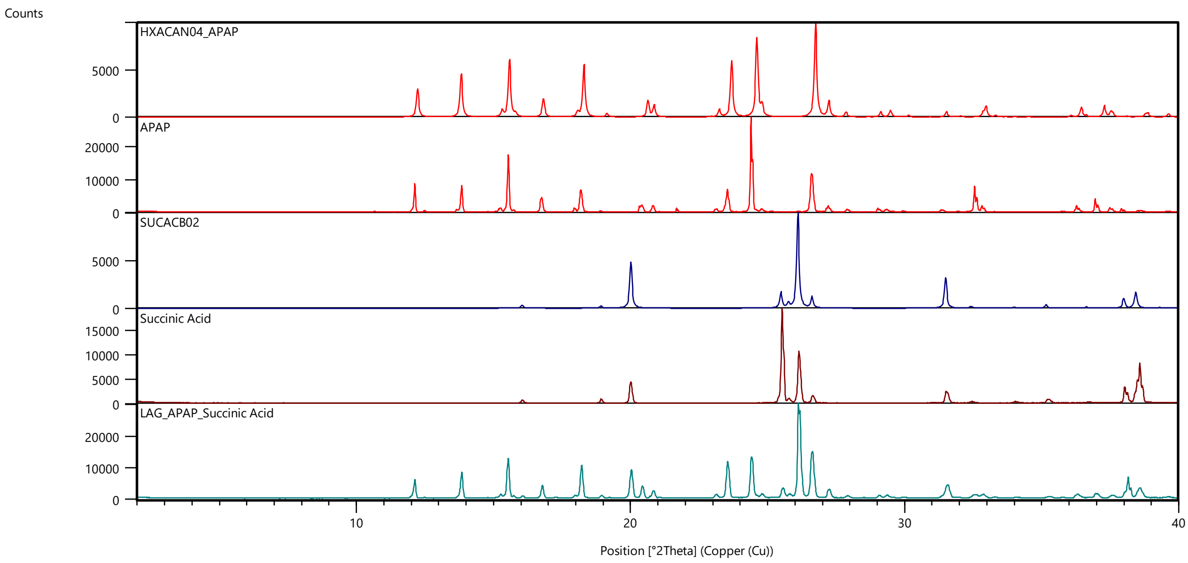

Figure S8. Comparison of experimental and from single crystal structure data simulated PXRD patterns of paracetamol (red), **succinic acid** and a physical mixture obtained from LAG grinding experiments.

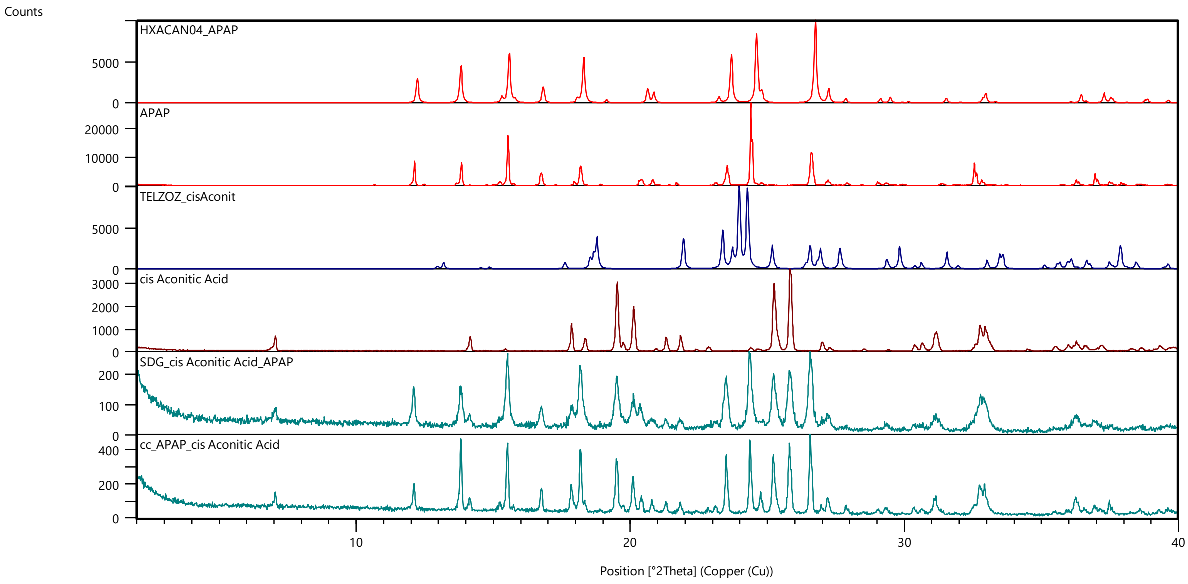

Figure S9. Comparison of experimental and from single crystal structure data simulated PXRD patterns of paracetamol (red), **cis-aconitic acid** and a physical mixture obtained from LAG grinding and crystallisation experiments.

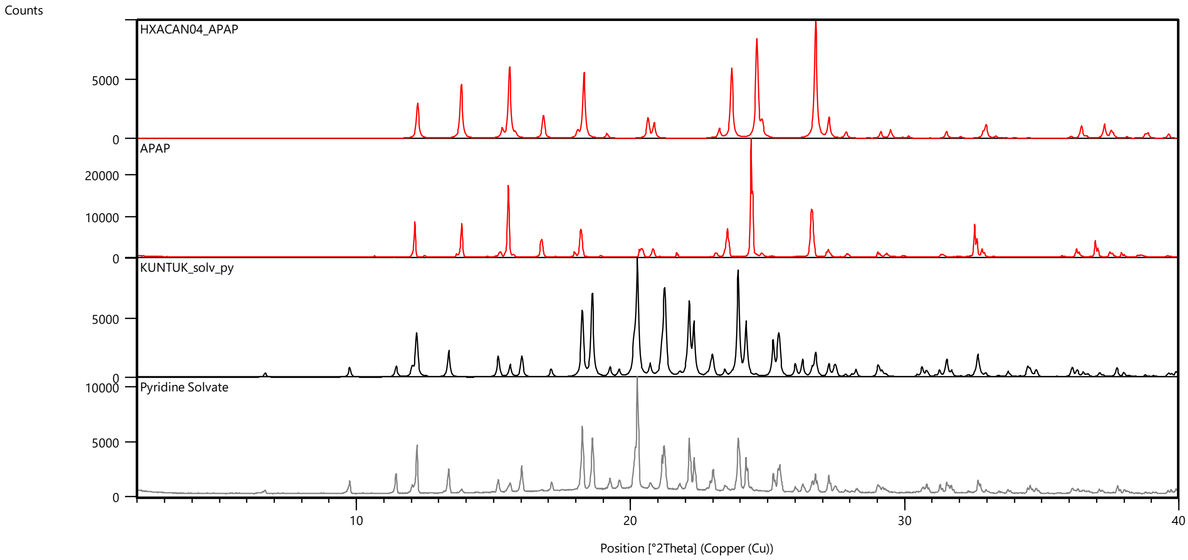

Figure S10. Comparison of experimental and from single crystal structure data simulated PXRD patterns of paracetamol (red) and the **pyridine solvate** of APAP.

2.3 Acetylsalicylic Acid (ASPI) cocrystal screen

No multicomponent forms were obtained using the chosen coformers/solvents (Table S2, Figure S11 - Figure S19).

Table S2. Overview acetylsalicylic acid crystallisation results.

Coformer	Contact Preparation	Slurry experiments			Liquid-assisted grinding		Co-sublimation
		n-Heptane	Pyridine	Diethyl ether	*n*-heptane	Diethyl ether	
Pyridoxine	x	x	n. a.	x	x	x	x
Methyl parabene	x	x	n. a.	x	x	x	x
Propyl parabene	x	x	n. a.	x	x	x	x
t-Butyl-4-hydroxyanisole	n. a.	x	n. a.	x	x	x	x
Nicotinic Acid	n. a.	x	n. a.	x	x	x	x
Nictoinamide	x	x	n. a.	x	x	x	x
Oxalic Acid	n. a.	x	n. a.	x	x	x	x
Succinic Acid	n. a.	x	n. a.	x	x	x	x
cis-Aconitic Acid	x	x	n. a.	x	x	x	x
Pyridine	n. a.	n. a.	x	n. a.	n. a.	n. a.	n. a.

n.a. – not attempted, x – physical mixture of the two compounds.

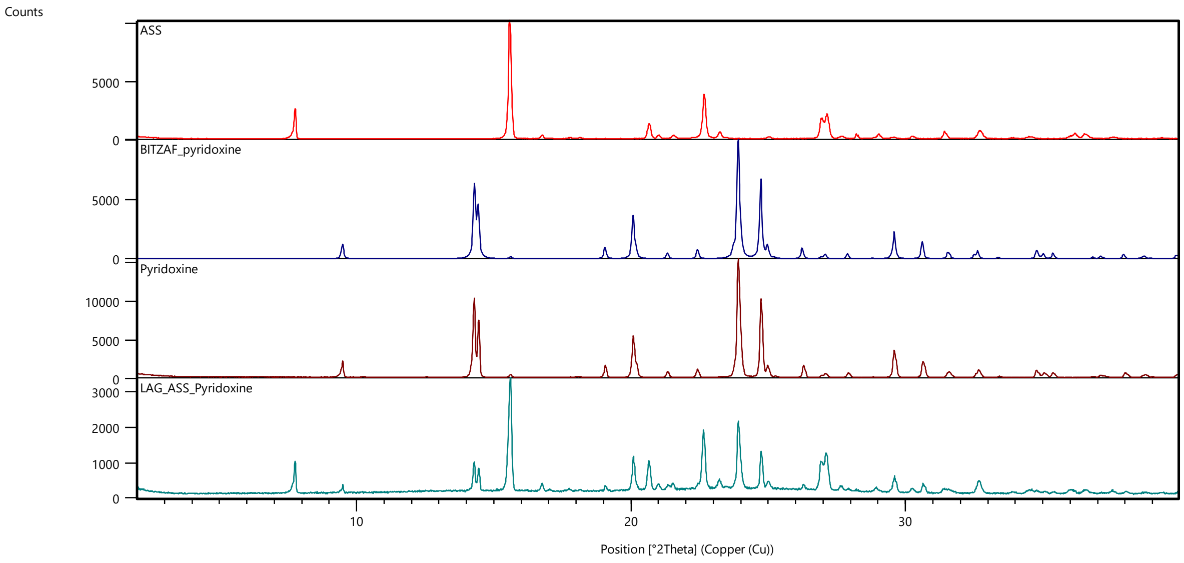

Figure S11. Comparison of experimental and from single crystal structure data simulated PXRD patterns of acetylsalicylic acid (red), **pyridoxine** and a physical mixture obtained from LAG grinding experiments.

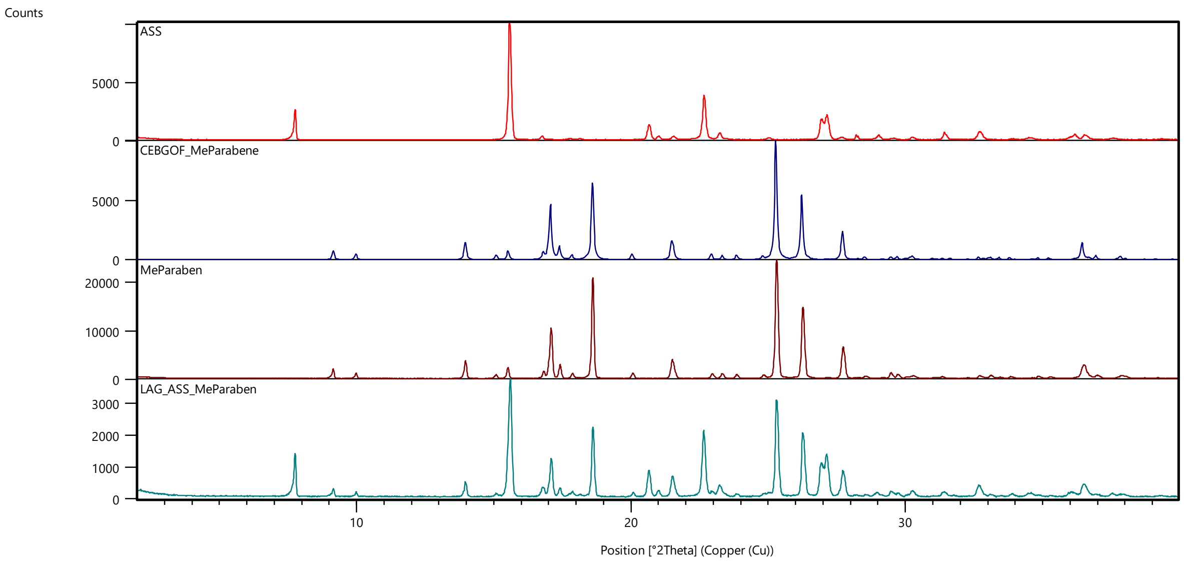

Figure S12. Comparison of experimental and from single crystal structure data simulated PXRD patterns of acetylsalicylic acid (red), **methyl paraben** and a physical mixture obtained from LAG grinding experiments.

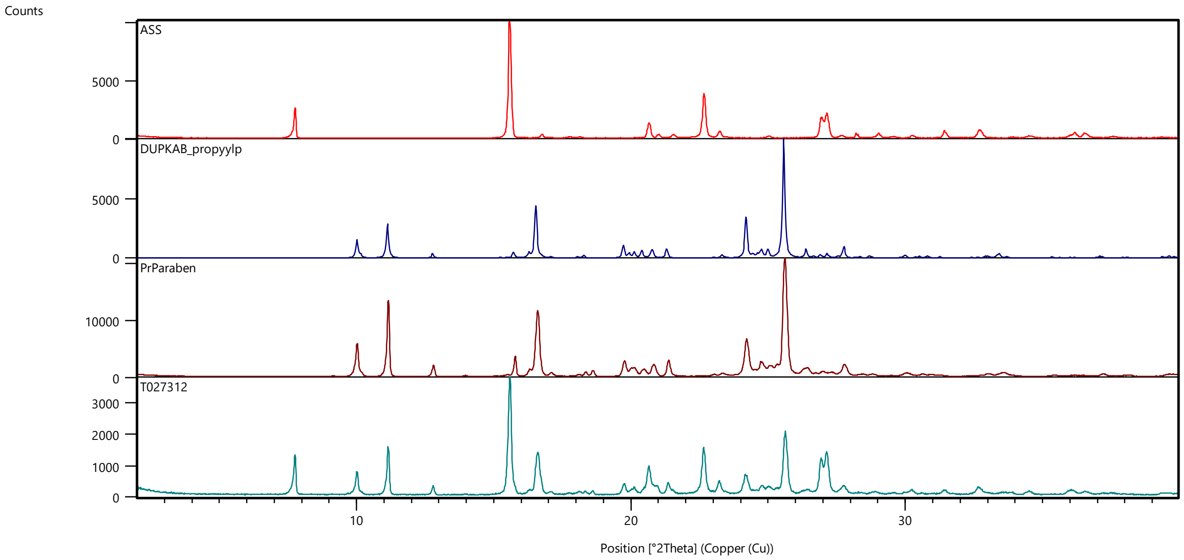

Figure S13. Comparison of experimental and from single crystal structure data simulated PXRD patterns of acetylsalicylic acid (red), **propyl paraben** and a physical mixture obtained from LAG grinding experiments.

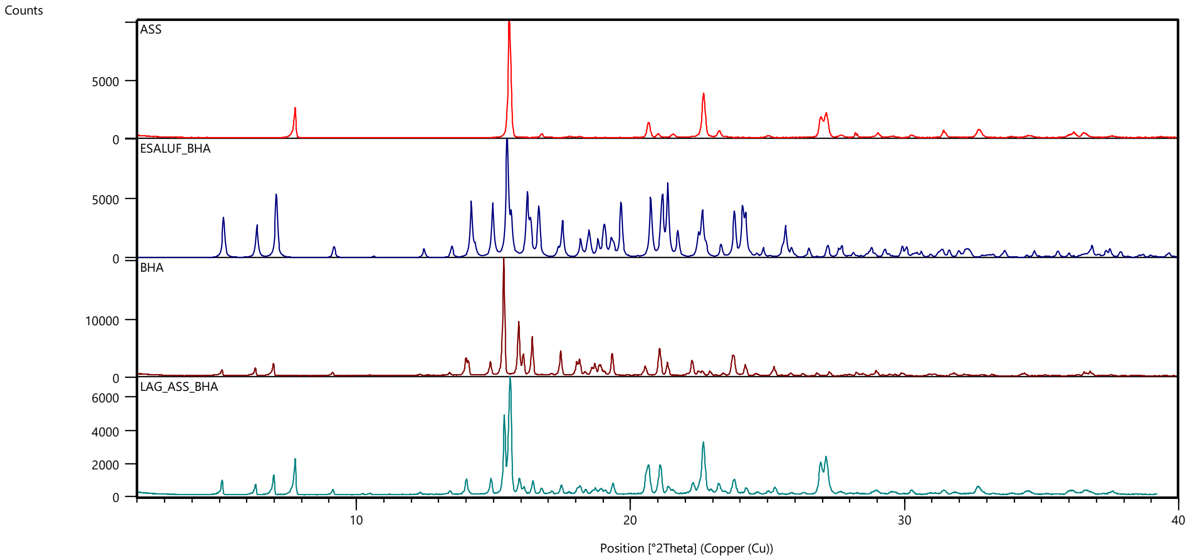

Figure S14. Comparison of experimental and from single crystal structure data simulated PXRD patterns of acetylsalicylic acid (red), **t-butyl-4-hydroxyanisole** and a physical mixture obtained from LAG grinding experiments.

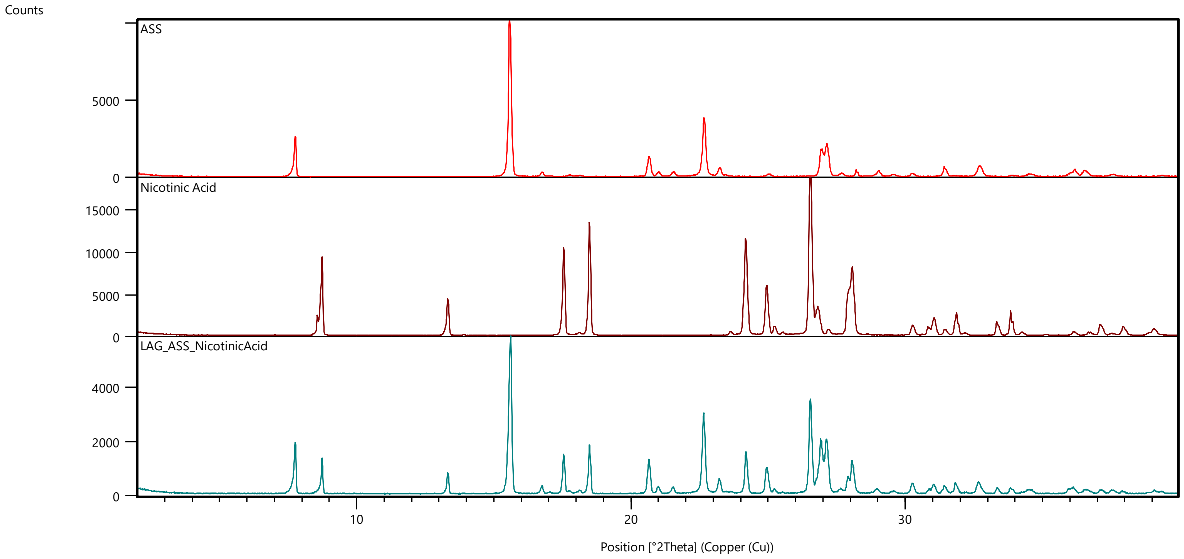

Figure S15. Comparison of experimental PXRD patterns of acetylsalicylic acid (red), **nicotinic acid** and a physical mixture obtained from LAG grinding experiments.

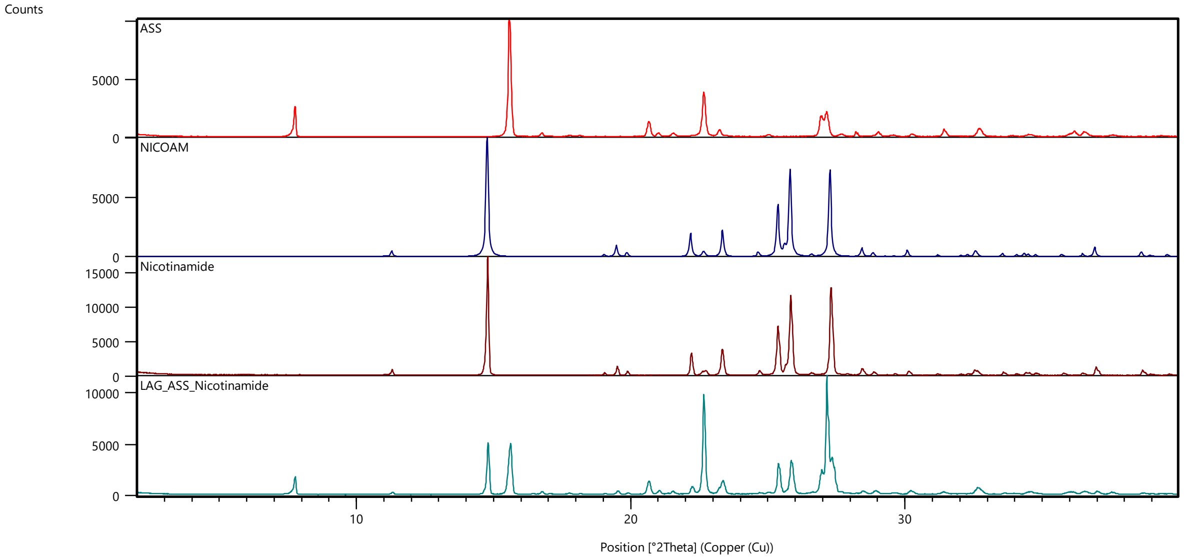

Figure S16. Comparison of experimental and from single crystal structure data simulated PXRD patterns of **acetylsalicylic acid** (red), nicotinamide and a physical mixture obtained from LAG grinding experiments.

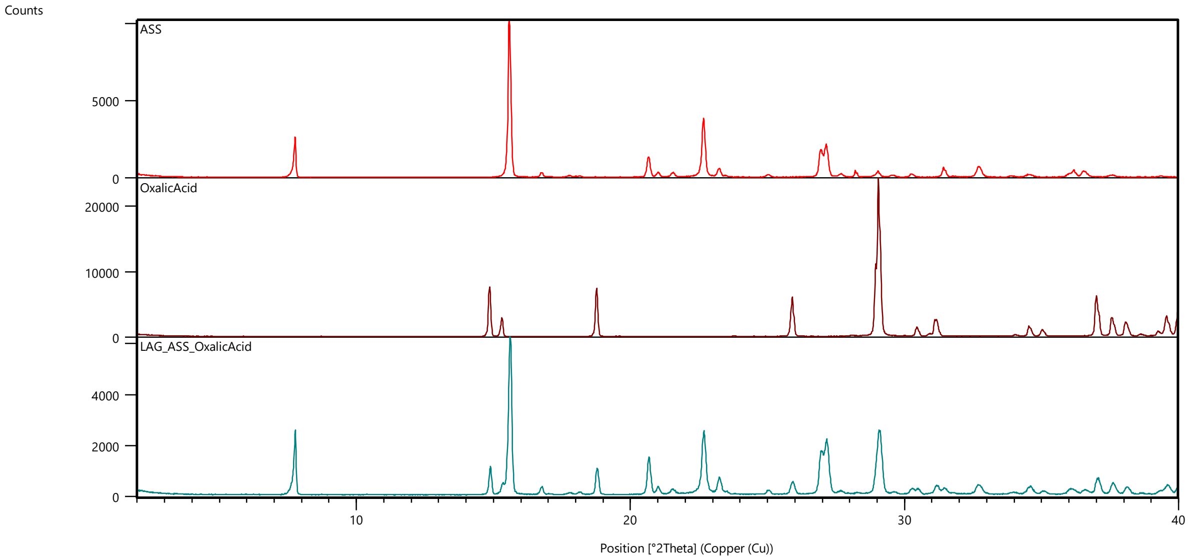

Figure S17. Comparison of experimental PXRD patterns of acetylsalicylic acid (red), **oxalic acid** and a physical mixture obtained from LAG grinding experiments.

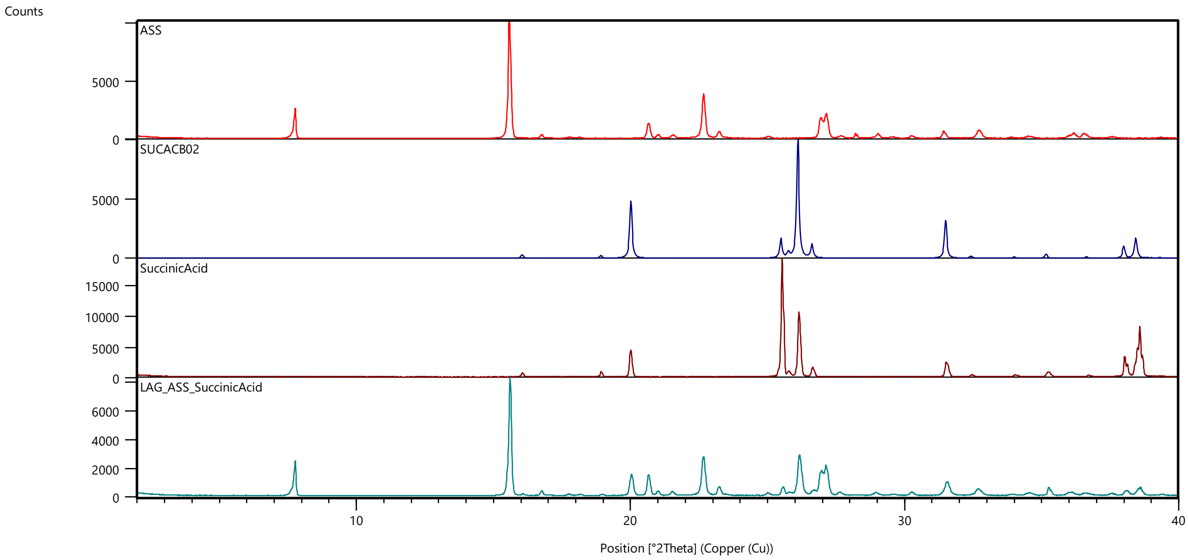

Figure S18. Comparison of experimental and from single crystal structure data simulated PXRD patterns of acetylsalicylic acid (red), **succinic aicd** and a physical mixture obtained from LAG grinding experiments.

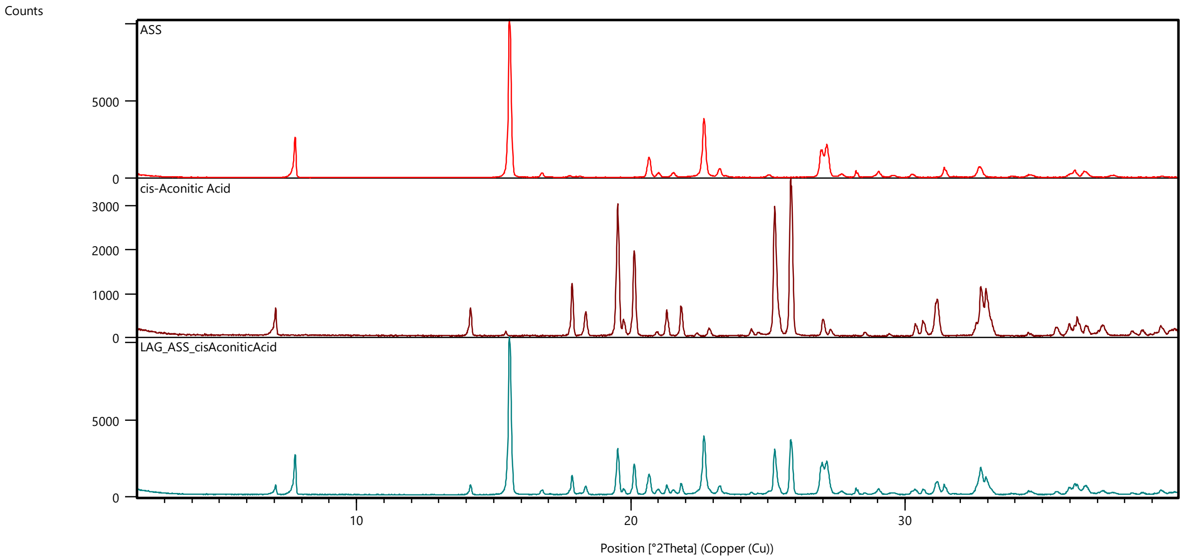

Figure S19. Comparison of experimental PXRD patterns of acetylsalicylic acid (red), **cis-aconitic aicd** and a physical mixture obtained from LAG grinding experiments.

2.4 Carbamazepine (CARB) cocrystal screen

All **experimental cocrystals** (nicotinamide, oxalic acid, succinic acid) were **reproduced** in the experimental screen. Furthermore, new cocrystals were found with methyl parabene, t-butyl-4-hydroxyanisole and cis-aconitic acid (Table S3, Figure S21 - Figure S29). In case of propyl paraben one experiment resulted in a new pattern.

Table S3. Overview carbamazepine crystallization results.

Coformer	Contact Preparation	Slurry experiments			Liquid-assisted grinding		Co-sublimation
		n-Heptane	Pyridine	Diethyl ether	*n*-heptane	Diethyl ether	
Pyridoxine	x	x	n. a.	x	x	x	x
Methyl parabene	yes	yes	n. a.	yes	yes	yes	x
Propyl parabene	x	Inconclusive	n. a.	x	x	x	x
t-Butyl-4-hydroxyanisole	n. a.	yes	n. a.	yes	yes	yes	x
Nicotinic Acid	n. a.	x	n. a.	x	x	x	x
Nictoinamide	yes	yes	n. a.	yes	yes	yes	x
Oxalic Acid	n. a.	yes	n. a.	yes	yes	yes	x
Succinic Acid	n. a.	yes	n. a.	yes	yes	yes	x
cis-Aconitic Acid	x	yes	n. a.	yes	yes	yes	x
Pyridine	n. a.	n. a.	x	n. a.	n. a.	n. a.	n. a.

n.a. – not attempted, x – physical mixture of the two compounds. Inconclusive refers to the ambiguous PXRD pattern observed in Figure S22

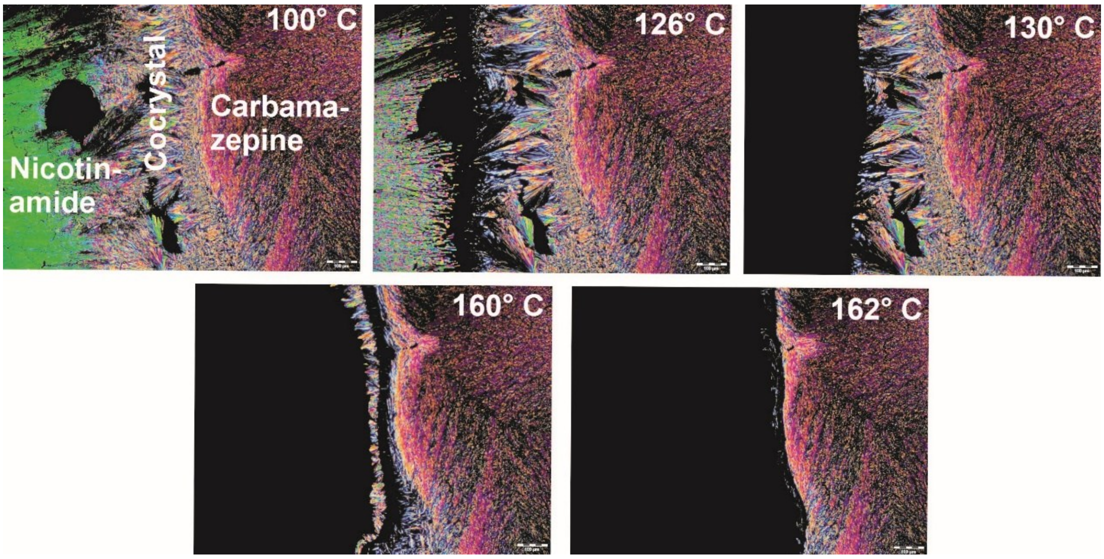

Figure S20. Contact preparation of nicotinamide-cocrystal-carbamazepine. At 126 °C and 160 °C the eutectic temperatures between nicotinamide and the cocrystal and carbamazepine and the cocrystal, respectively, can be seen.

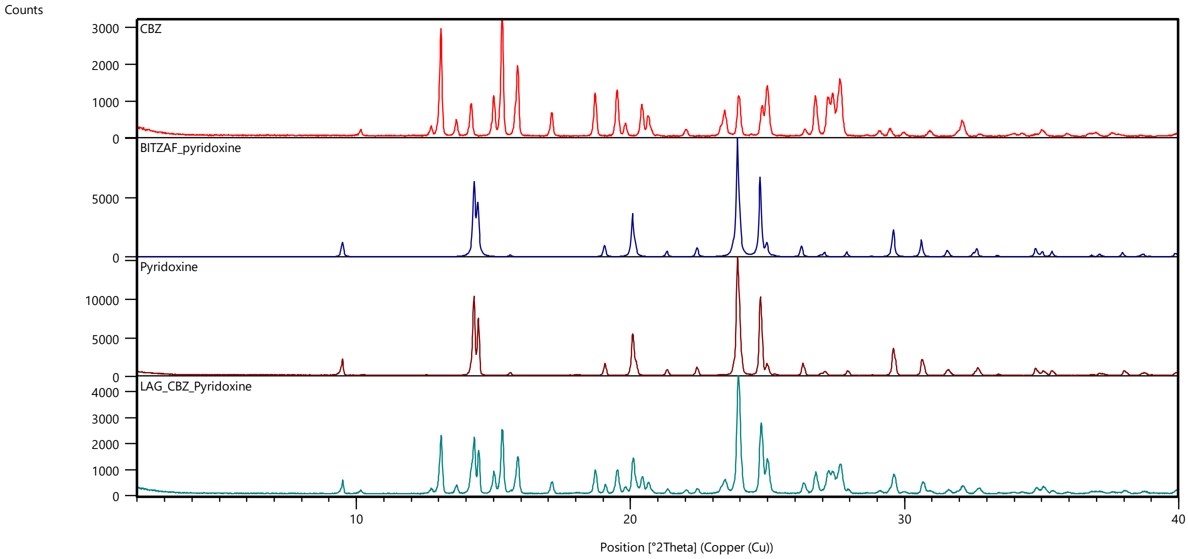

Figure S21. Comparison of experimental and from single crystal structure data simulated PXRD patterns of carbamazepine (red), **pyridoxine** and a physical mixture obtained from LAG grinding experiments.

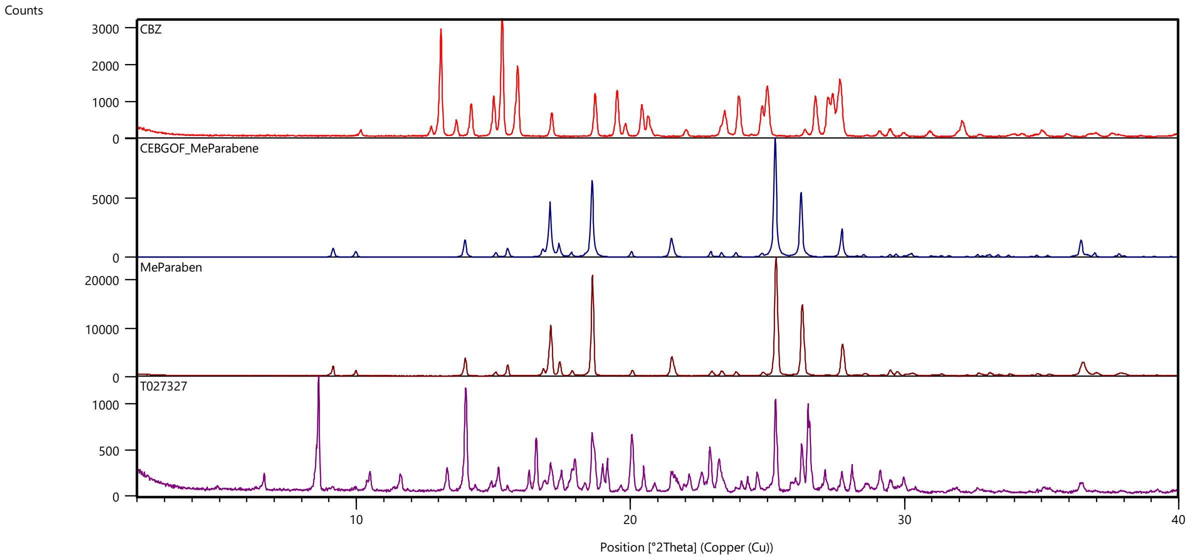

Figure S22. Comparison of experimental and from single crystal structure data simulated PXRD patterns of carbamazepine (red), **methyl parabene** and a cocrystal.

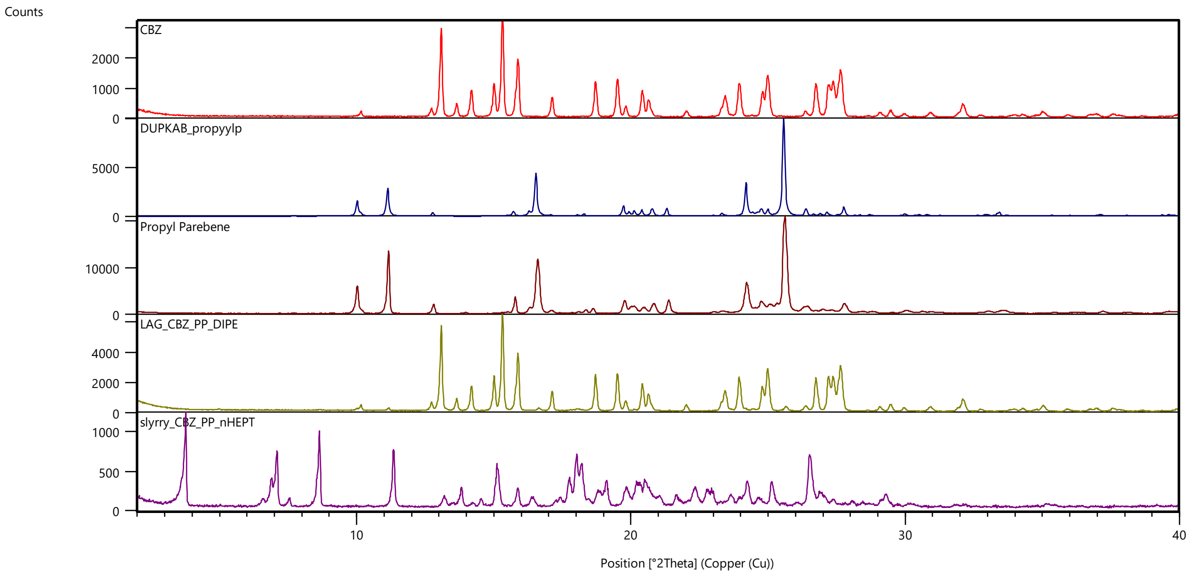

Figure S23. Comparison of experimental and from single crystal structure data simulated PXRD patterns of carbamazepine (red), **propyl parabene** and a physical mixture obtained from LAG grinding experiments.

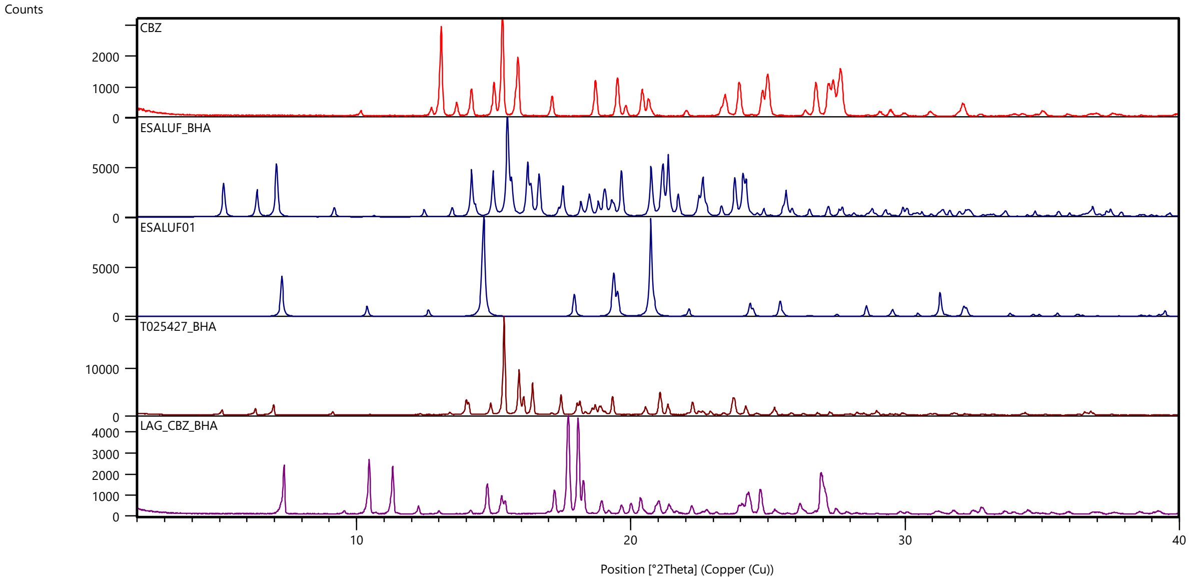

Figure S24. Comparison of experimental and from single crystal structure data simulated PXRD patterns of carbamazepine (red), **t-butyl-4-hydroxyanisole** and a cocrystal.

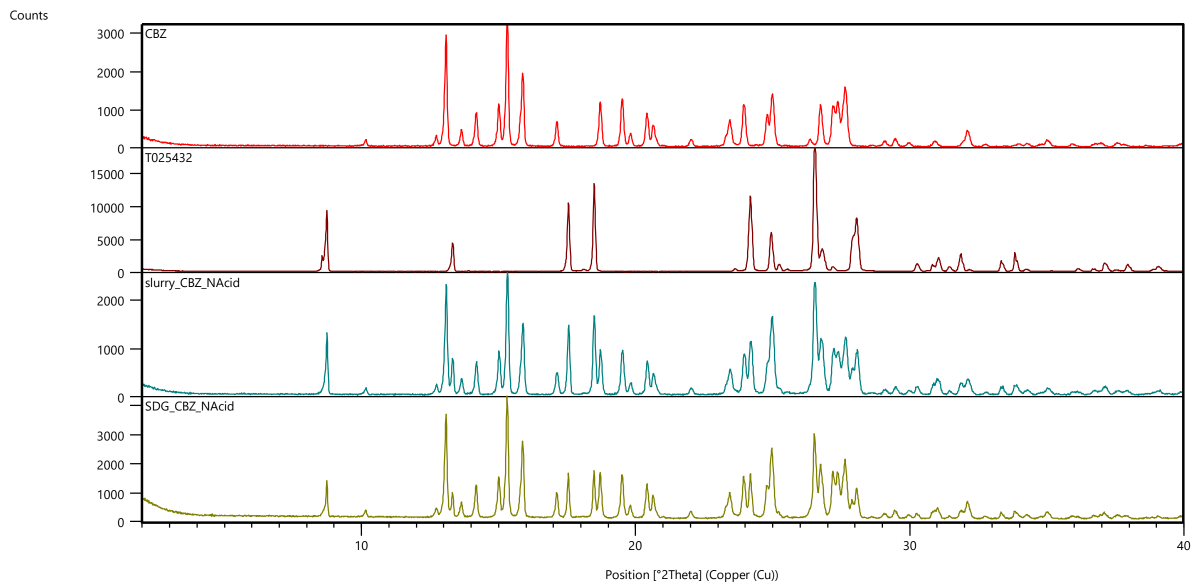

Figure S25. Comparison of experimental and from single crystal structure data simulated PXRD patterns of carbamazepine (red), **nicotinic acid** and a physical mixture obtained from LAG grinding/slurry experiments.

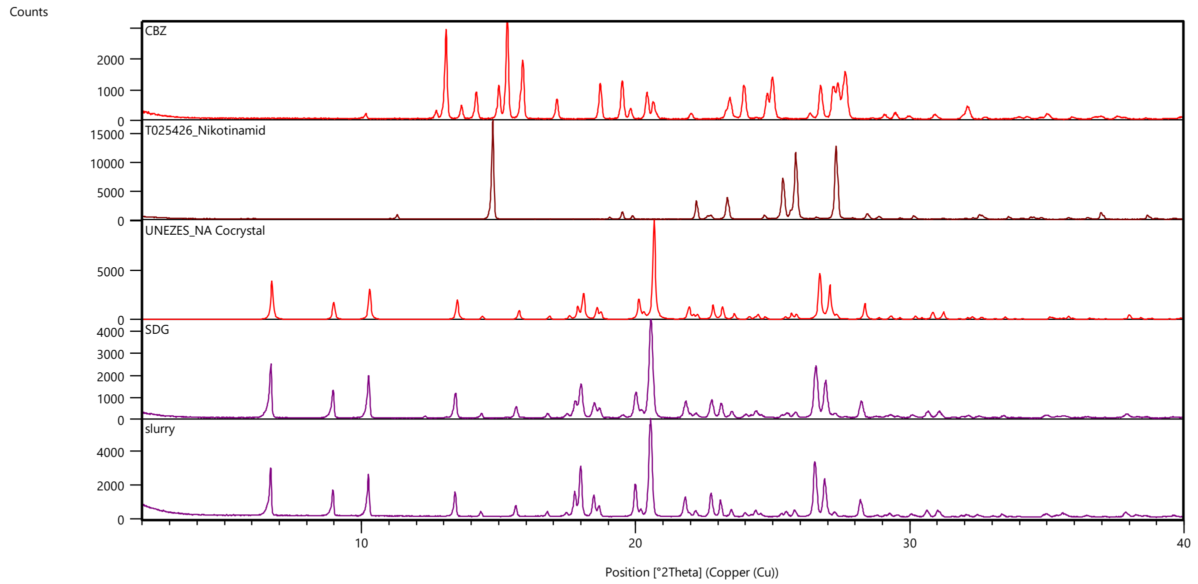

Figure S26. Comparison of experimental and from single crystal structure data simulated PXRD patterns of carbamazepine (first), **nicotinamide** and a cocrystal (SDG and slurry).

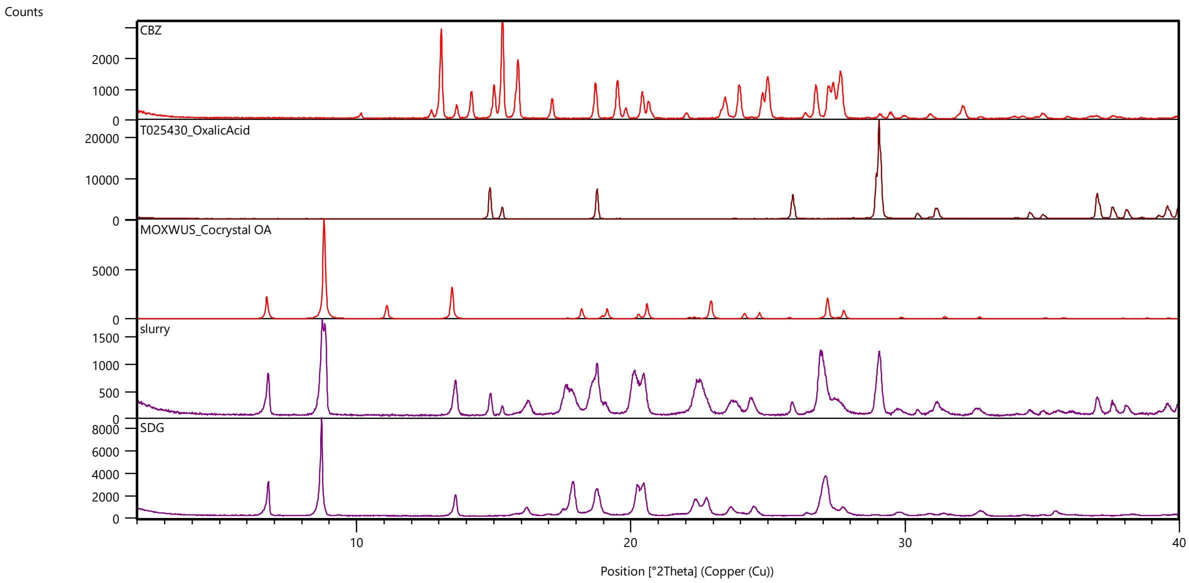

Figure S27. Comparison of experimental and from single crystal structure data simulated PXRD patterns of carbamazepine (first), **oxalic acid** and a cocrystal (SDG and slurry).

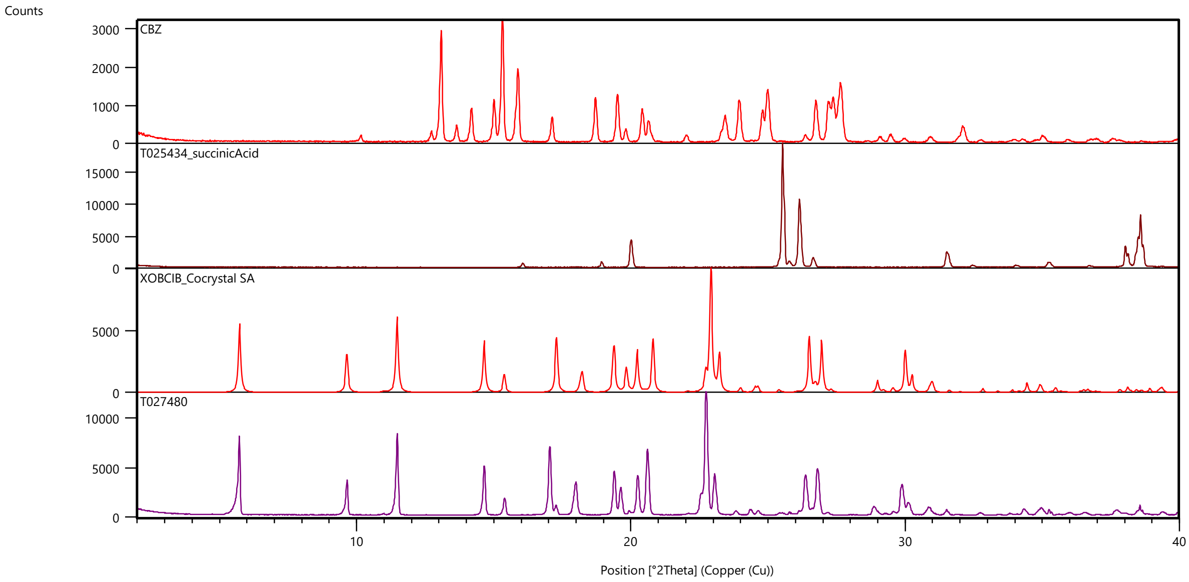

Figure S28. Comparison of experimental and from single crystal structure data simulated PXRD patterns of carbamazepine (first), **succinic acid** and a cocrystal (slurry experiment).

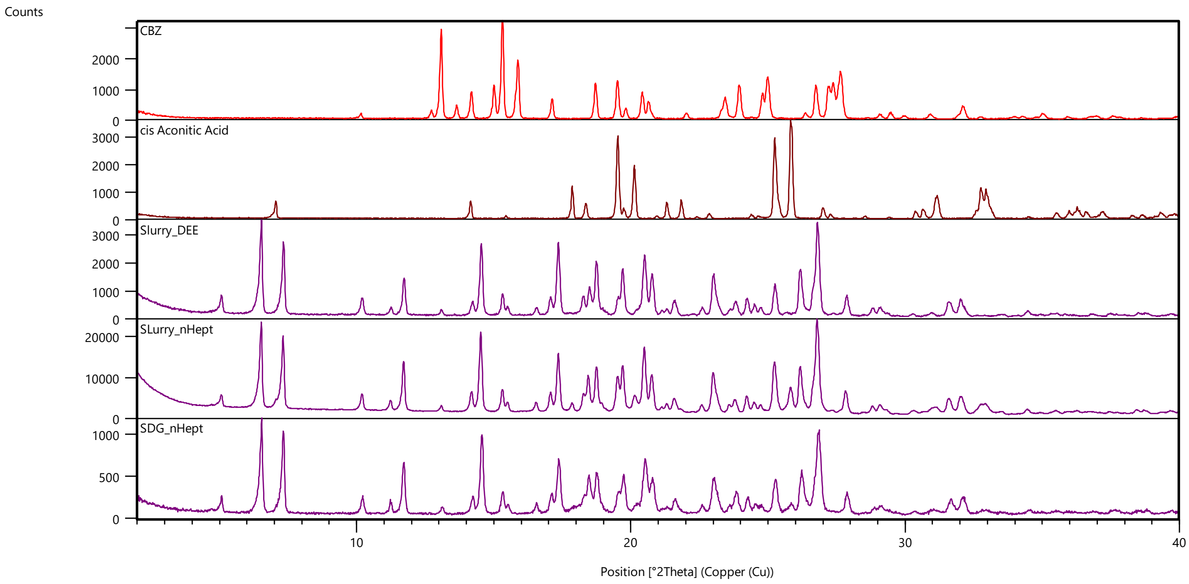

Figure S29. Comparison of experimental and from single crystal structure data simulated PXRD patterns of carbamazepine (first), **cis aconitic acid** and a cocrystal (SDG and slurry).

Additional cocrystallization experiments were undertaken for the combination **carbamazepine** and **propyl parabene** (Table S4) with the aim to reproduce the phase seen in the initial *n*-heptane slurry experiments (**Figure S23**). Therefore, the range of solvents, molar ratios and crystallisation techniques was extended. None of the additional experiments resulted in a cocrystal.

Table S4. Overview additional carbamazepine and propyl paraben cocrystallization results.

Solvent / molar ratio (CBZ:PP)	1:1	2:1	1:2
Grinding (dry or liquid-assisted)			
dry	x	x	x
dichloromethane	x, x[a]	x	x
dichloroethane	x	x	x
diethyl ether	x	x	x
diisopropyl ether	x	x	x
acetone	x	x	x
methanol	x	x	x
n-butanol	x	x	x
ethyl acetate	x	x	x
n-heptane	x	x	x
Slurry experimentes (10 °C – 30 °C)			
Diethyl ether	x	n. a.	n. a.
diisopropyl ether	x	n. a.	n. a.
n-butanol	x	n. a.	n. a.
n-heptane	x, x[a]	n. a.	n. a.
Solvent evaporation experiments (RT)			
acetone	x	n. a.	n. a.
methanol	x	n. a.	n. a.
ethanol	x	n. a.	n. a.
n-butanol	x	n. a.	n. a.
ethyl acetate	x	n. a.	n. a.
acetonitrile	x	n. a.	n. a.
Cooling crystallisation experiments			
acetone	x	n. a.	n. a.
methanol	x	n. a.	n. a.
ethanol	x	n. a.	n. a.
n-butanol	x	n. a.	n. a.
ethyl acetate	x	n. a.	n. a.
acetonitrile	x	n. a.	n. a.

n.a. – not attempted, x – physical mixture of the two compounds. [a]Two additional peak positions, otherwise physical mixture.

2.5 Rietveld refinements

DFT-d calculations (fixed cell and full optimization) were carried out with the CASTEP plane wave[2] code using the Perdew-Burke-Ernzerhof (PBE) generalized gradient approximation (GGA) exchange-correlation density functional and ultrasoft pseudopotentials, with the addition of the Tkatchenko and Scheffler (TS) semi-empirical dispersion corrections.[20] Brillouin zone integrations were performed on a symmetrized Monkhorst–Pack k-point grid with the number of *k*-points chosen to provide a maximum spacing of 0.07 $Å^{-1}$ and a basis set cut-off of 560 eV. The self-consistent field convergence on total energy was set to $1x10^{-5}$ eV per atom. Energy minimizations were performed using the Broyden–Fletcher–Goldfarb–Shanno optimisation scheme within the space group constraints. The optimizations were considered complete when energies were converged to better than $2x10^{-5}$ eV per atom, atomic displacements converged to $1x10^{-3}$ Å, maximum forces to $5x10^{-2}$ eV $Å^{-1}$, and maximum stresses were converged to $1x10^{-1}$ GPa.

Carbamazepine: 3-t-butyl-4-hydroxyanisole BHA cocrystals (CARB:ESAL-A)

The fixed cell PBE-TS structure was used as the starting point for rigid body Rietveld refinements in TOPAS academic.[3] The final refinements included a total of 54 parameters (28 profile, 4 cell, 1 scale, 1 isotropic temperature factor, 6 position and 6 rotation, 8 preferred orientation) yielding a final R_{wp}= 8.35%.

TITL **CARB:ESAL-A**
CELL 0.71073 9.5920 23.9589 10.6474 90 110.029 90
ZERR 4 0.0004 0.0007 0.0003 0 0.002 0
LATT 1
SYMM 1/2-x,1/2+y,1/2-z
SFAC C H N O
UNIT 104 112 8 12
FVAR 1.00
O1 O Uiso 0.4853(5) -0.0337(3) 0.1643(10) 1.000 0.062(2)
N1 N Uiso 0.4724(5) 0.0564(4) 0.2301(9) 1.000 0.062(2)
N2 N Uiso 0.2621(5) -0.0007(3) 0.1612(8) 1.000 0.062(2)
C1 C Uiso 0.3951(6) 0.0993(4) 0.2734(9) 1.000 0.062(2)
C2 C Uiso 0.4092(5) 0.0052(3) 0.1843(9) 1.000 0.062(2)
O2 O Uiso 0.342(3) 0.3804(6) 0.346(3) 1.000 0.062(2)
O3 O Uiso 0.902(2) 0.3649(8) 0.308(3) 1.000 0.062(2)
C3 C Uiso 0.6289(5) 0.0634(4) 0.2600(11) 1.000 0.062(2)
C4 C Uiso 0.2887(6) 0.1316(3) 0.1789(10) 1.000 0.062(2)
C5 C Uiso 0.4247(8) 0.1071(4) 0.4117(9) 1.000 0.062(2)
C6 C Uiso 0.7245(6) 0.0614(5) 0.3943(12) 1.000 0.062(2)
C7 C Uiso 0.6845(5) 0.0713(4) 0.1563(12) 1.000 0.062(2)
C8 C Uiso 0.2061(7) 0.1709(3) 0.2199(10) 1.000 0.062(2)
C9 C Uiso 0.3376(9) 0.1463(4) 0.4503(10) 1.000 0.062(2)
C10 C Uiso 0.5423(8) 0.0780(5) 0.5144(9) 1.000 0.062(2)
C11 C Uiso 0.6717(7) 0.0583(5) 0.5067(10) 1.000 0.062(2)
C12 C Uiso 0.8785(6) 0.0647(5) 0.4197(13) 1.000 0.062(2)
C13 C Uiso 0.8370(5) 0.0760(4) 0.1843(14) 1.000 0.062(2)

C14 C Uiso 0.2302(9) 0.1779(4) 0.3560(10) 1.000 0.062(2)
C15 C Uiso 0.9340(5) 0.0722(5) 0.3162(15) 1.000 0.062(2)
C16 C Uiso 0.691(2) 0.3169(7) 0.330(3) 1.000 0.062(2)
C17 C Uiso 0.763(2) 0.3667(7) 0.314(3) 1.000 0.062(2)
C18 C Uiso 0.548(2) 0.3219(6) 0.337(3) 1.000 0.062(2)
C19 C Uiso 0.768(2) 0.2597(7) 0.347(3) 1.000 0.062(2)
C20 C Uiso 0.479(3) 0.3735(6) 0.333(3) 1.000 0.062(2)
C21 C Uiso 0.692(3) 0.4183(7) 0.308(3) 1.000 0.062(2)
C22 C Uiso 0.902(2) 0.2594(8) 0.479(3) 1.000 0.062(2)
C23 C Uiso 0.6649(19) 0.2121(7) 0.355(2) 1.000 0.062(2)
C24 C Uiso 0.822(2) 0.2465(7) 0.229(3) 1.000 0.062(2)
C25 C Uiso 0.552(3) 0.4221(6) 0.318(3) 1.000 0.062(2)
C26 C Uiso 0.263(2) 0.3314(6) 0.359(3) 1.000 0.062(2)
H1 H Uiso 0.2197(5) -0.0401(3) 0.1443(8) 1.000 0.074(3)
H2 H Uiso 0.2012(5) 0.0307(3) 0.1793(7) 1.000 0.074(3)
H3 H Uiso 0.2708(6) 0.1253(3) 0.0733(10) 1.000 0.074(3)
H4 H Uiso 0.6076(6) 0.0722(3) 0.0537(12) 1.000 0.074(3)
H5 H Uiso 0.1224(8) 0.1955(3) 0.1453(11) 1.000 0.074(3)
H6 H Uiso 0.3565(10) 0.1517(5) 0.5566(10) 1.000 0.074(3)
H7 H Uiso 0.5290(10) 0.0762(6) 0.6119(9) 1.000 0.074(3)
H8 H Uiso 0.7522(8) 0.0425(6) 0.5991(11) 1.000 0.074(3)
H9 H Uiso 0.9540(6) 0.0628(6) 0.5228(14) 1.000 0.074(3)
H10 H Uiso 0.8798(6) 0.0822(4) 0.1028(15) 1.000 0.074(3)
H11 H Uiso 0.1640(10) 0.2078(4) 0.3885(11) 1.000 0.074(3)
H12 H Uiso 1.0534(5) 0.0757(5) 0.3391(16) 1.000 0.074(3)
H13 H Uiso 0.941(3) 0.4036(8) 0.312(3) 1.000 0.074(3)
H14 H Uiso 0.491(2) 0.2841(6) 0.348(3) 1.000 0.074(3)
H15 H Uiso 0.750(3) 0.4561(7) 0.297(3) 1.000 0.074(3)
H16 H Uiso 0.958(2) 0.2186(9) 0.493(3) 1.000 0.074(3)
H17 H Uiso 0.865(2) 0.2660(9) 0.565(3) 1.000 0.074(3)
H18 H Uiso 0.983(2) 0.2920(9) 0.480(3) 1.000 0.074(3)
H19 H Uiso 0.626(2) 0.2164(7) 0.440(2) 1.000 0.074(3)
H20 H Uiso 0.5677(19) 0.2082(6) 0.264(2) 1.000 0.074(3)
H21 H Uiso 0.7262(18) 0.1727(7) 0.369(2) 1.000 0.074(3)
H22 H Uiso 0.8656(19) 0.2037(7) 0.239(3) 1.000 0.074(3)
H23 H Uiso 0.910(2) 0.2751(8) 0.226(3) 1.000 0.074(3)
H24 H Uiso 0.730(2) 0.2493(7) 0.133(3) 1.000 0.074(3)
H25 H Uiso 0.501(3) 0.4628(6) 0.316(3) 1.000 0.074(3)
H26 H Uiso 0.326(2) 0.3072(6) 0.449(3) 1.000 0.074(3)
H27 H Uiso 0.159(2) 0.3456(6) 0.368(3) 1.000 0.074(3)
H28 H Uiso 0.239(2) 0.3047(5) 0.270(3) 1.000 0.074(3)
END

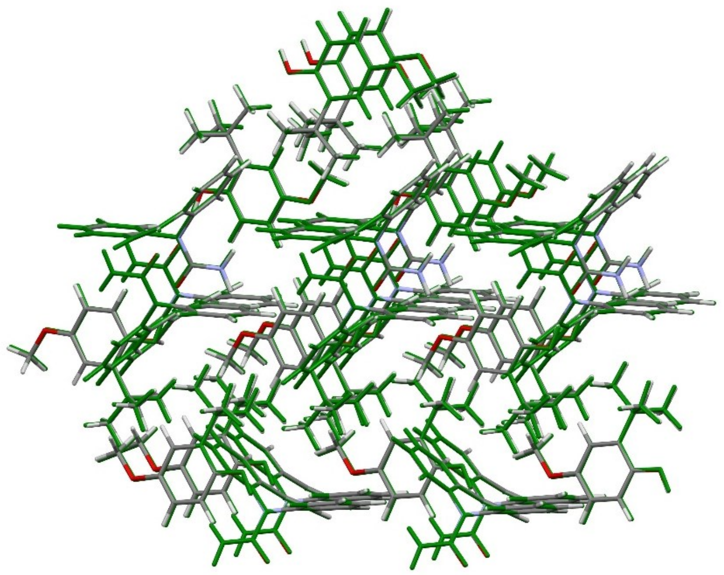

Figure S30. Overlay of the 30-molecule cluster of the observed structure of **CARB:ESAL-A** (colored by element, main disorder component only) and calculated PBE-TS structure (green, cell parameters fixed, atom positions optimized), $rmsd_{30}$=0.07 Å.

Carbamazepine: 3-t-butyl-4-hydroxyanisole BHA cocrystals (CARB:ESAL-B)

The PBE-TS structure (fixed cell parameters) were used as the starting point for rigid body Rietveld refinements in TOPAS academic.[3] The final refinements included a total of 61 parameters (28 profile, 4 cell, 1 scale, 1 isotropic temperature factor, 6 position and 6 rotation, 15 preferred orientation) yielding a final R_{wp}= 6.56%.

TITL **CARB:ESAL-B**
CELL 0.71073 12.6807 7.7880 23.3619 90 96.927 90
ZERR 4 0.0005 0.0003 0.0010 0 0.002 0
LATT 1
SYMM -x,1/2+y,1/2-z
SFAC C H N O
UNIT 104 112 8 12
FVAR 1.00
O1 O Uiso 0.0278(12) 0.079(2) 0.2212(3) 1.000 0.076(4)
N1 N Uiso 0.2087(12) 0.073(3) 0.2390(3) 1.000 0.076(4)
N2 N Uiso 0.1116(13) 0.289(3) 0.2778(3) 1.000 0.076(4)
C1 C Uiso 0.1886(11) -0.230(2) 0.2127(4) 1.000 0.076(4)
C2 C Uiso 0.2054(10) -0.322(2) 0.1155(4) 1.000 0.076(4)
O2 O Uiso 0.8398(12) 0.688(4) 0.0029(14) 1.000 0.076(4)
O3 O Uiso 0.8505(9) 0.124(4) 0.1485(13) 1.000 0.076(4)
C3 C Uiso 0.1851(11) -0.360(2) 0.1717(4) 1.000 0.076(4)
C4 C Uiso 0.2315(9) -0.156(2) 0.1011(4) 1.000 0.076(4)
C5 C Uiso 0.2388(9) -0.022(2) 0.1422(4) 1.000 0.076(4)
C6 C Uiso 0.2788(9) 0.145(2) 0.1275(4) 1.000 0.076(4)
C7 C Uiso 0.3359(10) 0.258(2) 0.1635(5) 1.000 0.076(4)
C8 C Uiso 0.3671(11) 0.242(3) 0.2253(5) 1.000 0.076(4)
C9 C Uiso 0.4613(12) 0.321(3) 0.2504(6) 1.000 0.076(4)
C10 C Uiso 0.4969(14) 0.302(3) 0.3087(6) 1.000 0.076(4)

C11 C Uiso 0.4373(15) 0.208(3) 0.3443(5) 1.000 0.076(4)
C12 C Uiso 0.3420(14) 0.133(3) 0.3211(4) 1.000 0.076(4)
C13 C Uiso 0.3074(12) 0.149(3) 0.2622(4) 1.000 0.076(4)
C14 C Uiso 0.2128(11) -0.062(2) 0.1979(3) 1.000 0.076(4)
C15 C Uiso 0.1115(12) 0.146(2) 0.2455(3) 1.000 0.076(4)
C16 C Uiso 0.9249(13) 0.809(4) 0.0089(15) 1.000 0.076(4)
C17 C Uiso 0.7588(10) 0.444(4) 0.0388(13) 1.000 0.076(4)
C18 C Uiso 0.8473(11) 0.553(4) 0.0412(14) 1.000 0.076(4)
C19 C Uiso 0.9358(10) 0.519(4) 0.0810(15) 1.000 0.076(4)
C20 C Uiso 0.9350(9) 0.375(4) 0.1164(14) 1.000 0.076(4)
C21 C Uiso 0.8477(9) 0.265(4) 0.1133(13) 1.000 0.076(4)
C22 C Uiso 0.7555(9) 0.299(4) 0.0742(13) 1.000 0.076(4)
C23 C Uiso 0.6554(8) 0.186(4) 0.0719(12) 1.000 0.076(4)
C24 C Uiso 0.5648(8) 0.254(3) 0.0278(11) 1.000 0.076(4)
C25 C Uiso 0.6132(9) 0.185(4) 0.1312(11) 1.000 0.076(4)
C26 C Uiso 0.6803(7) 0.001(4) 0.0549(12) 1.000 0.076(4)
H1 H Uiso 0.0425(14) 0.354(3) 0.2807(4) 1.000 0.091(4)
H2 H Uiso 0.1798(14) 0.342(3) 0.2970(4) 1.000 0.091(4)
H3 H Uiso 0.1707(13) -0.256(3) 0.2564(4) 1.000 0.091(4)
H4 H Uiso 0.2008(10) -0.423(2) 0.0830(5) 1.000 0.091(4)
H5 H Uiso 0.1650(12) -0.490(2) 0.1835(5) 1.000 0.091(4)
H6 H Uiso 0.2487(8) -0.128(2) 0.0574(4) 1.000 0.091(4)
H7 H Uiso 0.2709(8) 0.176(2) 0.0815(4) 1.000 0.091(4)
H8 H Uiso 0.3699(10) 0.368(2) 0.1435(5) 1.000 0.091(4)
H9 H Uiso 0.5074(12) 0.397(3) 0.2231(7) 1.000 0.091(4)
H10 H Uiso 0.5710(14) 0.362(3) 0.3265(7) 1.000 0.091(4)
H11 H Uiso 0.4643(16) 0.195(4) 0.3902(5) 1.000 0.091(4)
H12 H Uiso 0.2929(15) 0.062(3) 0.3484(4) 1.000 0.091(4)
H13 H Uiso 0.9191(9) 0.122(4) 0.1743(14) 1.000 0.091(4)
H14 H Uiso 0.9324(14) 0.872(4) 0.0515(15) 1.000 0.091(4)
H15 H Uiso 0.9047(15) 0.906(4) -0.0247(15) 1.000 0.091(4)
H16 H Uiso 1.0009(13) 0.749(4) 0.0019(16) 1.000 0.091(4)
H17 H Uiso 0.6904(10) 0.477(4) 0.0081(13) 1.000 0.091(4)
H18 H Uiso 1.0053(11) 0.602(4) 0.0850(15) 1.000 0.091(4)
H19 H Uiso 1.0044(10) 0.347(4) 0.1472(15) 1.000 0.091(4)
H20 H Uiso 0.4951(8) 0.170(3) 0.0284(11) 1.000 0.091(4)
H21 H Uiso 0.5404(9) 0.385(3) 0.0381(11) 1.000 0.091(4)
H22 H Uiso 0.5859(8) 0.254(3) -0.0165(11) 1.000 0.091(4)
H23 H Uiso 0.5956(10) 0.316(4) 0.1445(11) 1.000 0.091(4)
H24 H Uiso 0.6698(9) 0.128(4) 0.1650(12) 1.000 0.091(4)
H25 H Uiso 0.5392(9) 0.111(3) 0.1286(11) 1.000 0.091(4)
H26 H Uiso 0.6080(7) -0.078(4) 0.0529(11) 1.000 0.091(4)
H27 H Uiso 0.7084(7) -0.005(4) 0.0123(12) 1.000 0.091(4)
H28 H Uiso 0.7410(8) -0.059(4) 0.0860(12) 1.000 0.091(4)
END

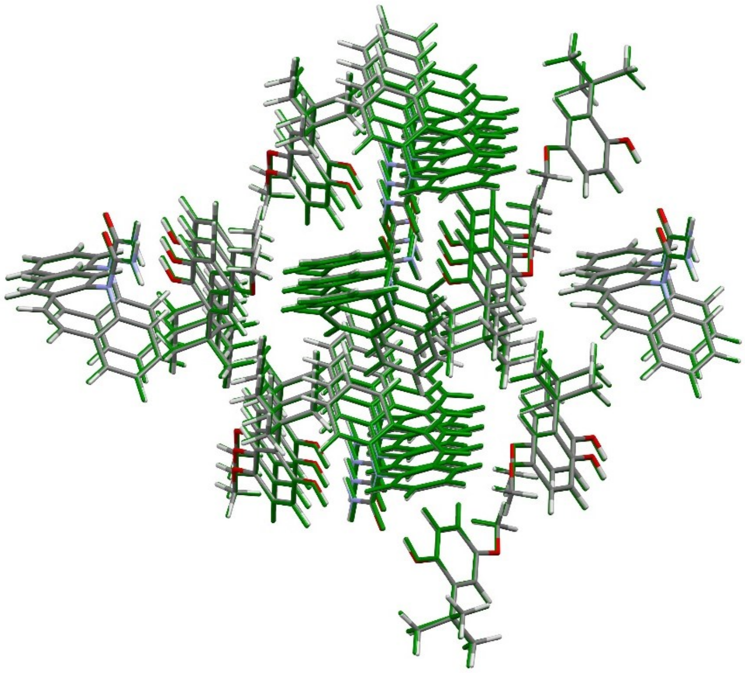

Figure S31. Overlay of the 30-molecule cluster of the observed structure of **CARB:ESAL-B** (colored by element, main disorder component only) and calculated PBE-TS structure (green, cell parameters fixed, atom positions optimized), $rmsd_{30}$=0.08 Å.

Carbamazepine:methyl paraben CEBG cocrystals (CARB-CEBG-A)

The fixed cell PBE-TS structure was used as the starting point for rigid body Rietveld refinements in TOPAS academic.[3] The final refinements included a total of 54 parameters (26 profile, 6 cell, 1 scale, 1 isotropic temperature factor, 6 position and 6 rotation, 8 preferred orientation) yielding a final R_{wp}= 7.62%.

TITL **CARB-CEBG-A**
CELL 0.71073 6.6607 8.4157 17.7876 89.651 87.906 87.519
ZERR 2 0.0002 0.0003 0.0006 0.003 0.003 0.004
LATT 1
SFAC C H N O
UNIT 46 40 4 8
FVAR 1.00
O1 O Uiso 0.6188(17) -0.201(2) 0.2401(8) 1.000 0.076(4)
N1 N Uiso 0.7326(15) 0.019(2) 0.1784(8) 1.000 0.076(4)
N2 N Uiso 0.6140(19) 0.035(3) 0.3037(8) 1.000 0.076(4)
C1 C Uiso 0.6826(12) -0.126(2) 0.0617(8) 1.000 0.076(4)
C2 C Uiso 0.9581(13) -0.279(3) 0.0018(10) 1.000 0.076(4)
O2 O Uiso 0.558(11) 0.126(5) 0.571(2) 1.000 0.076(4)
O3 O Uiso 0.362(10) 0.599(4) 0.299(2) 1.000 0.076(4)
C3 C Uiso 0.7560(13) -0.227(2) 0.0042(9) 1.000 0.076(4)
O4 O Uiso 0.871(11) 0.198(6) 0.534(2) 1.000 0.076(4)
C4 C Uiso 1.0863(13) -0.227(3) 0.0556(10) 1.000 0.076(4)
C5 C Uiso 1.0169(13) -0.123(3) 0.1136(10) 1.000 0.076(4)
C6 C Uiso 1.1568(14) -0.068(3) 0.1680(10) 1.000 0.076(4)
C7 C Uiso 1.1487(16) 0.073(3) 0.2053(10) 1.000 0.076(4)
C8 C Uiso 1.0000(17) 0.204(3) 0.1981(10) 1.000 0.076(4)

C9 C Uiso 1.056(2) 0.362(3) 0.2065(10) 1.000 0.076(4)
C10 C Uiso 0.921(2) 0.490(3) 0.1935(10) 1.000 0.076(4)
C11 C Uiso 0.726(2) 0.462(2) 0.1712(9) 1.000 0.076(4)
C12 C Uiso 0.6652(17) 0.306(2) 0.1652(8) 1.000 0.076(4)
C13 C Uiso 0.7994(16) 0.178(3) 0.1800(9) 1.000 0.076(4)
C14 C Uiso 0.8106(13) -0.077(3) 0.1167(9) 1.000 0.076(4)
C15 C Uiso 0.6538(17) -0.056(2) 0.2420(8) 1.000 0.076(4)
C16 C Uiso 0.952(11) 0.113(7) 0.598(2) 1.000 0.076(4)
C17 C Uiso 0.598(10) 0.299(5) 0.465(2) 1.000 0.076(4)
C18 C Uiso 0.669(11) 0.199(5) 0.528(2) 1.000 0.076(4)
C19 C Uiso 0.395(10) 0.296(4) 0.4458(19) 1.000 0.076(4)
C20 C Uiso 0.724(10) 0.404(5) 0.425(2) 1.000 0.076(4)
C21 C Uiso 0.649(10) 0.503(5) 0.369(2) 1.000 0.076(4)
C22 C Uiso 0.445(10) 0.501(4) 0.351(2) 1.000 0.076(4)
C23 C Uiso 0.320(10) 0.394(4) 0.3893(19) 1.000 0.076(4)
H1 H Uiso 0.550(2) -0.017(3) 0.3506(8) 1.000 0.091(4)
H2 H Uiso 0.648(2) 0.152(3) 0.3051(8) 1.000 0.091(4)
H3 H Uiso 0.5250(12) -0.086(2) 0.0657(7) 1.000 0.091(4)
H4 H Uiso 1.0152(15) -0.361(3) -0.0419(10) 1.000 0.091(4)
H5 H Uiso 0.6555(13) -0.266(2) -0.0384(8) 1.000 0.091(4)
H6 H Uiso 1.2442(13) -0.268(3) 0.0535(11) 1.000 0.091(4)
H7 H Uiso 1.2904(15) -0.146(3) 0.1754(11) 1.000 0.091(4)
H8 H Uiso 1.2757(17) 0.098(3) 0.2400(11) 1.000 0.091(4)
H9 H Uiso 1.208(2) 0.386(3) 0.2226(11) 1.000 0.091(4)
H10 H Uiso 0.966(3) 0.611(3) 0.2012(11) 1.000 0.091(4)
H11 H Uiso 0.623(2) 0.563(2) 0.1606(9) 1.000 0.091(4)
H12 H Uiso 0.5125(16) 0.281(2) 0.1505(8) 1.000 0.091(4)
H13 H Uiso 0.462(10) 0.676(4) 0.278(2) 1.000 0.091(4)
H14 H Uiso 0.896(11) 0.169(7) 0.650(2) 1.000 0.091(4)
H15 H Uiso 1.115(11) 0.122(7) 0.591(3) 1.000 0.091(4)
H16 H Uiso 0.914(11) 0.025(6) 0.597(2) 1.000 0.091(4)
H17 H Uiso 0.298(10) 0.216(4) 0.4768(18) 1.000 0.091(4)
H18 H Uiso 0.880(10) 0.410(6) 0.440(2) 1.000 0.091(4)
H19 H Uiso 0.746(10) 0.587(5) 0.339(2) 1.000 0.091(4)
H20 H Uiso 0.163(10) 0.392(4) 0.3746(18) 1.000 0.091(4)
END

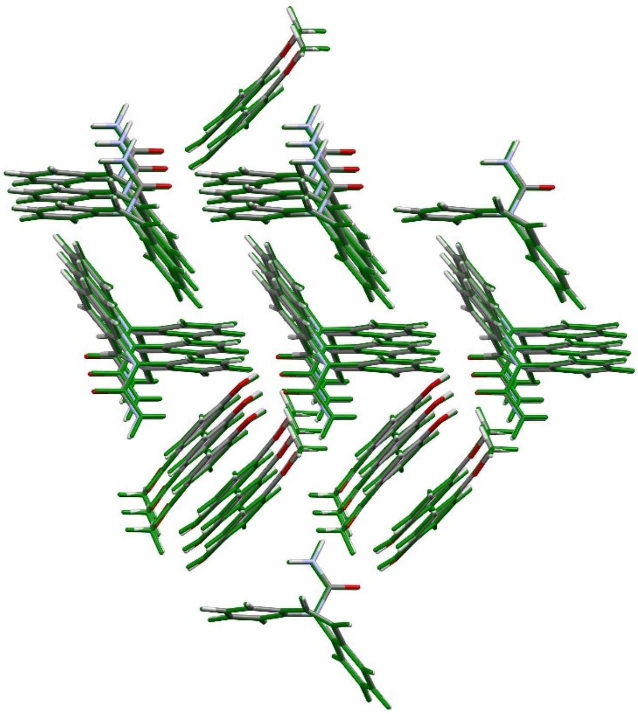

Figure S32. Overlay of the 30-molecule cluster of the observed structure of **CARB-CEBG-A** (colored by element, main disorder component only) and calculated PBE-TS structure (green, cell parameters fixed, atom positions optimized), $rmsd_{30}$=0.09 Å.

cis Aconitic acid (form II)

The PBE-TS structure (fixed cell parameters) were used as the starting point for rigid body Rietveld refinements in TOPAS academic.[3] The final refinements included a total of 58 parameters (38 profile, 4 cell, 1 scale, 1 isotropic temperature factor, 3 position and 3 rotation, 8 preferred orientation) yielding a final R_{wp}= 8.02%. Note that only one position of the likely disordered COOH function was refined.

TITL **cis Aconitic acid (form II)**
CELL 0.71073 25.2072 4.91846 11.5654 90 97.8134 90
ZERR 8 0.0008 0.00014 0.0004 0 0.0015 0
LATT 7
SYMM -x,y,1/2-z
SFAC C H O
UNIT 48 48 48
FVAR 1.00
O1 O Uiso 0.2531(2) 0.9545(14) 0.4151(6) 1.000 0.0414(14)
O2 O Uiso 0.1331(2) 0.8155(11) 0.4378(5) 1.000 0.0414(14)
O3 O Uiso 0.2015(3) 1.3319(12) 0.3931(6) 1.000 0.0414(14)
O4 O Uiso 0.08025(18) 0.5533(8) 0.3086(4) 1.000 0.0414(14)
O5 O Uiso 0.0450(2) 1.0372(6) 0.1224(5) 1.000 0.0414(14)
O6 O Uiso 0.03986(17) 0.7218(5) -0.0199(4) 1.000 0.0414(14)
C1 C Uiso 0.1862(2) 0.9939(10) 0.2485(6) 1.000 0.0414(14)
C2 C Uiso 0.11915(17) 0.7286(7) 0.1166(5) 1.000 0.0414(14)
C3 C Uiso 0.14327(19) 0.8300(9) 0.2343(5) 1.000 0.0414(14)
C4 C Uiso 0.2157(2) 1.0876(12) 0.3623(6) 1.000 0.0414(14)

C5 C Uiso 0.11900(19) 0.7359(9) 0.3379(4) 1.000 0.0414(14)
C6 C Uiso 0.06459(18) 0.8433(6) 0.0747(5) 1.000 0.0414(14)
H1 H Uiso 0.06791(18) 0.4772(9) 0.3800(4) 1.000 0.0498(16)
H2 H Uiso 0.00379(18) 0.8190(5) -0.0516(4) 1.000 0.0498(16)
H3 H Uiso 0.2033(2) 1.0697(10) 0.1726(6) 1.000 0.0498(16)
H4 H Uiso 0.14402(18) 0.7875(8) 0.0499(5) 1.000 0.0498(16)
H5 H Uiso 0.11660(15) 0.5053(7) 0.1146(4) 1.000 0.0498(16)
H6 H Uiso 0.2231(3) 1.3950(13) 0.4704(7) 1.000 0.0498(16)
END

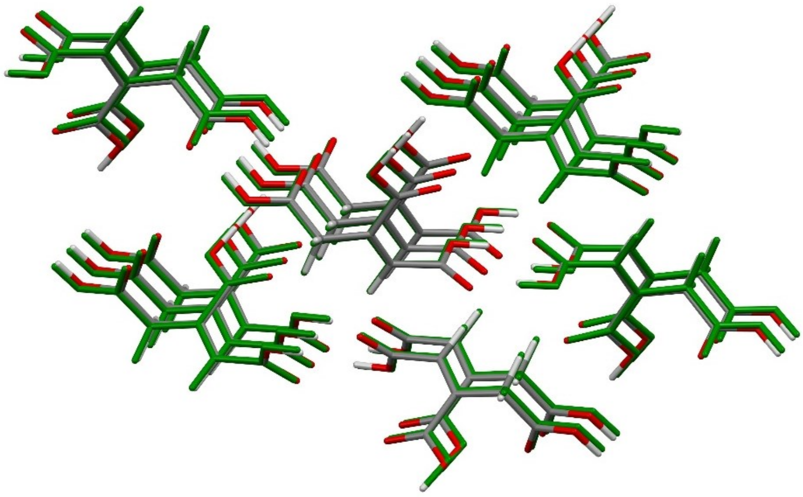

Figure S33. Overlay of the 15-molecule cluster of the observed structure of **cis Aconitic acid form II** (colored by element, main disorder component only) and calculated PBE-TS structure (green, cell parameters fixed, atom positions optimized), $rmsd_{15}$=0.14 Å.

References

1. Etter, M. C.; MacDonald, J. C.; Bernstein, J., Graph-set analysis of hydrogen-bond patterns in organic crystals. *Acta Crystallographica, Section B: Structural Science* **1990,** *B46* (2), 256-262.
2. Clark, S. J.; Segall, M. D.; Pickard, C. J.; Hasnip, P. J.; Probert, M. J.; Refson, K.; Payne, M. C., First principles methods using CASTEP. *Zeitschrift fur Kristallographie* **2005,** *220* (5-6), 567-570.
3. Coelho, A. A. *Topas Academic V5*, Coelho Software: Brisbane, 2012.
